# PLUSPULS: A transcranial magnetic stimulator with extended pulse protocols

**DOI:** 10.1016/j.ohx.2022.e00380

**Published:** 2022-12-09

**Authors:** Christoph Staat, Norbert Gattinger, Bernhard Gleich

**Affiliations:** Munich Institute of BioEngineering (MIBE) at Technical University of Munich (TUM) Boltzmannstra 11, 85748 Garching b. Muchen, Germany

**Keywords:** Transcranial magnetic stimulation, ppTMS, qTBS, Modular, Flexible, Biphasic

## Abstract

Transcranial magnetic stimulation (TMS) is increasingly applied in basic neuroscience while its field of usage for diagnosing and treating various neurological diseases broadens steadily. A TMS device generates a current pulse in the reach of several thousand ampére to produce a magnetic pulse which induces an electric field around neurons. This electric field, if high enough to depolarize the neuron membrane, generates an action potential at the neuron which travels down the neurons connected to it. The PLUSPULS TMS generates this magnetic pulse by pre-charging a pulse capacitor C with the voltage $V_{C0}$ and connecting it with a stimulation coil L. The oscillation of the resonance circuit is cut off after one period and is called a biphasic pulse. PLUSPULS is a high frequency stimulator with inter stimulus intervals (ISI) down to 1ms which enables different pulse protocols as paired pulse or quadri theta burst stimulation. A GUI on PC allows a flexible control of PLUSPULS with varying amplitudes and ISI in one burst. The modular hardware and the control via GUI on PC allows for an easier adjustment on requirements to come. The article provides design considerations, hardware, firmware and software to reconstruct a modular biphasic TMS with enhanced charging network to enable extended pulse protocols.

## Graphical abstract

**Figure:**
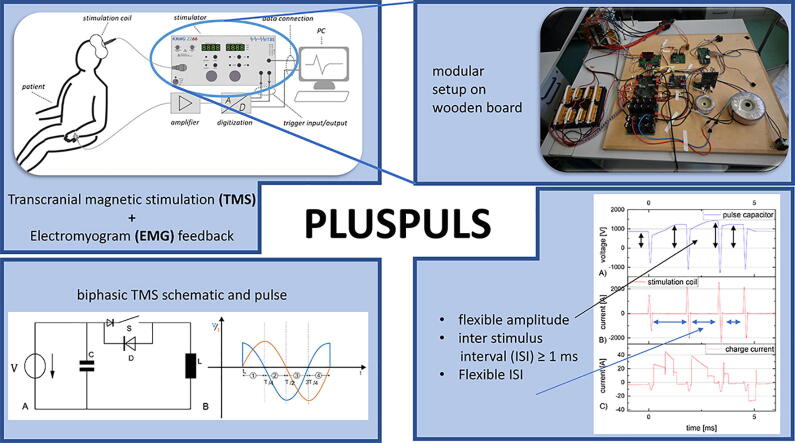

**Specifications table**

**Table:** 

**Hardware name**	*PLUSPULS*
**Subject area**	*Neuroscience*
**Hardware type**	*Electrical engineering and computer science*
**Closest commercial analog**	*POWERMAG MAG* & *More*
**Open source license**	*CERN-OHL-W-2.0*
**Cost of hardware**	*approx. 10000* €
**Source file repository**	https://doi.org/10.5281/zenodo.6457647
**OSHWA certification UID**	DE000124

## 1 Hardware in Context

PLUSPULS is a biphasic TMS device capable of different pulse protocols. Since TMS was first introduced by Barker et al. in 1985 [1], its areas of application have steadily been growing and becoming more and more differentiated, primarily due to the fact that TMS is pain-free and non-invasive. TMS can be applied as a mapping [2], diagnostic [3], [4], and treatment [5], [6] tool for different neurological diseases. TMS devices are approved for the treatment of depression [7] and obsessive compulsive disorder [8]. Further promising TMS applications are currently evaluated and discussed for stroke rehabilitation [9], [10], [11], [12], [13], [14], [15], [16], [17] and Parkinson’s disease [18].

TMS is supposed to change the plasticity [19], [20], [21], [22], [23], [24] and the metaplasticity [25], [26] of the brain. Since plasticity is the ability of the brain to adapt to new circumstances and represents the learning capability, it is a very interesting field of application for TMS. However, it is still investigated how the efficacy of plasticity induction can be increased [27], [25], [20].

TMS can be separated into different groups by pulse shape. The monophasic [1], the biphasic [28], [29] and other more complex TMS [30], [31], [32], [33] with more possibilities for the pulse shape.

### 1.1 Principle of TMS

In TMS, a pulsed current through a stimulation coil generates a pulsed magnetic field. The pulsed magnetic field in turn induces a pulsed electric field inside the brain that depolarizes neurons and thus evokes action potentials. A tangential position of the round or figure-of-eight stimulation coil as close as possible to the head is best to evoke an action potential, as the magnetic field strength of the two coil types, decreases rapidly with increased distance to the coil [34], [35]. The electric field has to be sufficient to depolarize the membrane potential in order to generate an action potential at the neuron. The action potential travels transsynaptically to other neurons. If motor cortical neurons are depolarized, it descends as a volley down the spinal cord to the peripheral neurons of the corresponding muscle.

**Fig. 1:**
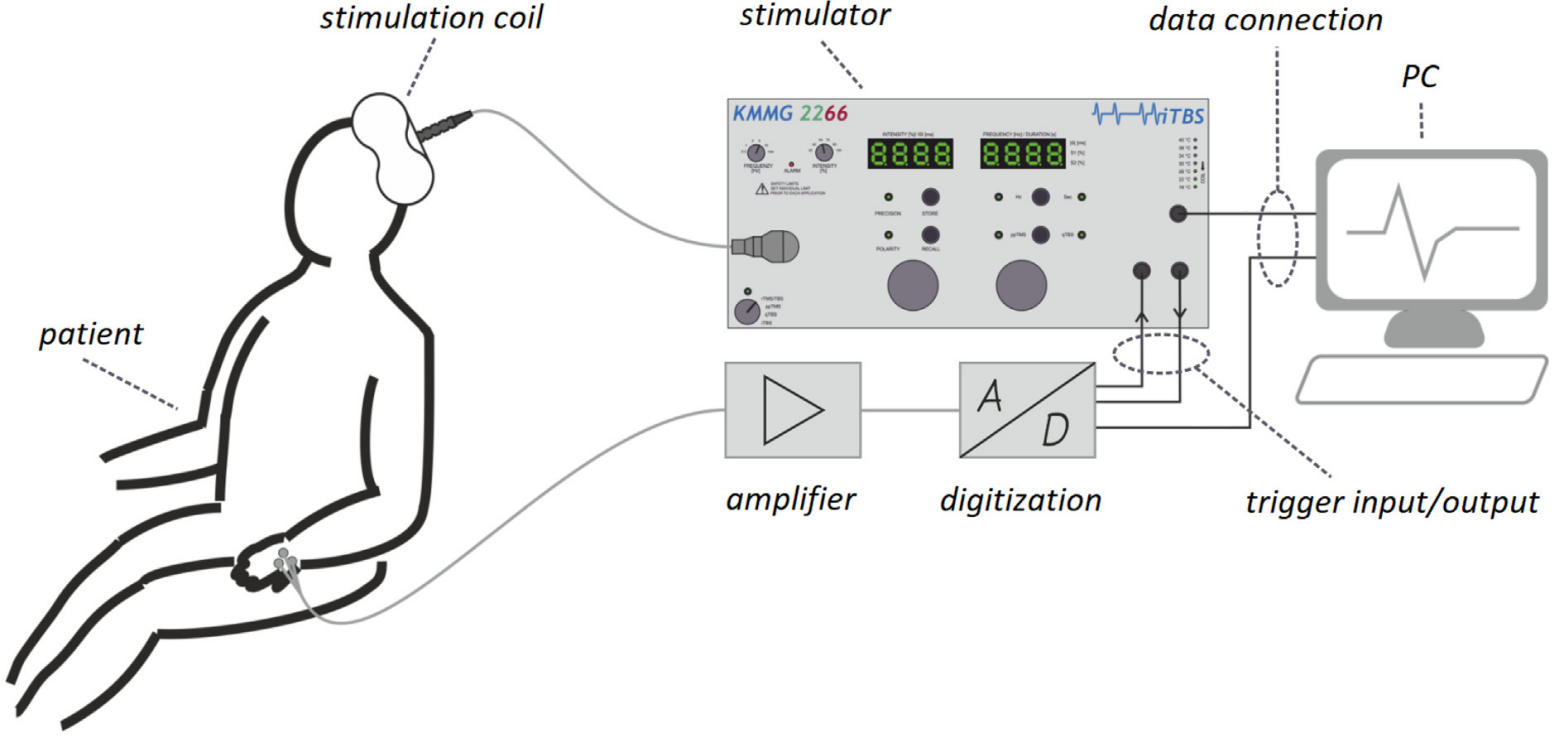
A TMS controlled via PC used for the stimulation of motor cortical neurons. The action potential generated by the stimulation travels down the spine to the corresponding muscle. The motor evoked potential (MEP) can be measured via elektromyography (EMG). The MEP is amplified, digitized and sent back to the PC.

A exemplary TMS session is illustrated in Fig. 1. The current pulse generated by the stimulator (Fig. 1) flows through the stimulation coil. The induced electric field is high enough to depolarize the membrane potential of motor cortical neurons to generate an action potential. This travels down the spinal cord to the corresponding muscle. The reaction of the muscle to the stimulation can be measured as motor evoked potential (MEP). The MEP is amplified, digitized and send to the computer. A change in the motor cortico-spinal neuron interactions can be detected if the same stimulation pulses generate a different MEP response.

Generating an MEP requires the stimulation amplitude to be higher than a certain threshold. These thresholds are the motor thresholds (MT). The corresponding MT for the MEP is usually expressed as a percentage of the maximum stimulator output (MSO) to generate a MEP in 50% of the cases [36]. The two relevant excitability thresholds are called resting motor threshold (RMT) and active motor threshold (AMT). Stimulating pulses and their amplitudes are often relating to the MT to calibrate the stimulator output. For example, a paired-pulse protocol can consist of one pulse with an amplitude lower than the MT, a so called sub-threshold pulse, and one pulse with an amplitude higher than the MT, a so called supra-threshold pulse [4].

### 1.2 PLUSPULS stimulation pulse

**Fig. 2:**
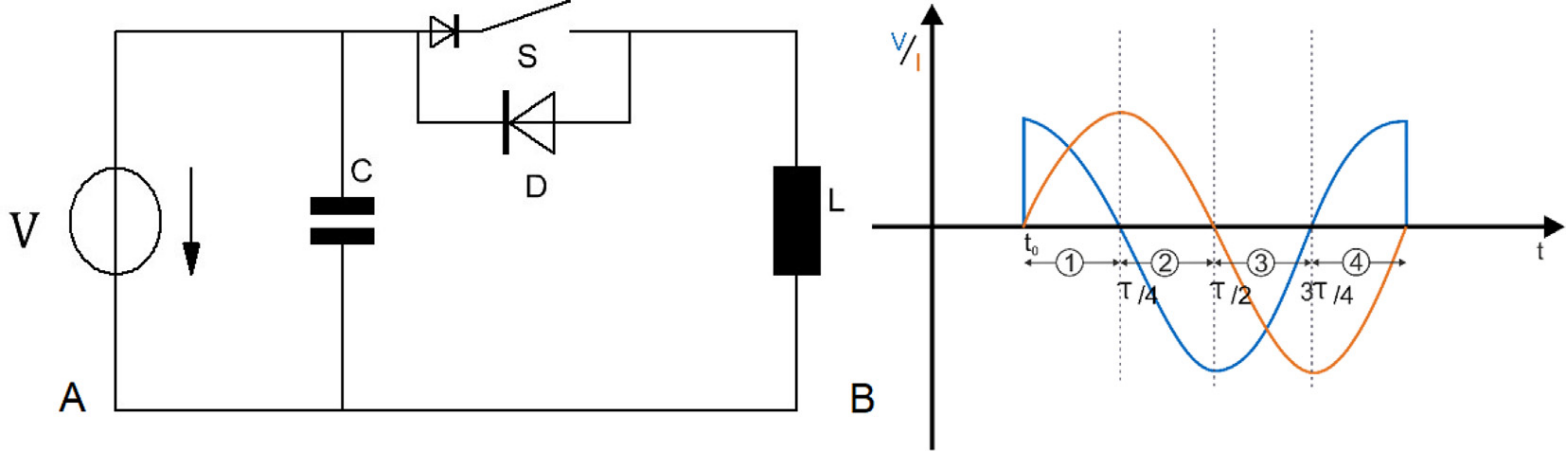
(A) A schematic for a biphasic TMS device that consits of the pulse capacitor C, the high voltage power supply V, a unipolar switch S, the antiparallel diode D and the stimulation coil L. (B) Current (orange) and voltage (blue) graphs for a biphasic pulse in the lossless case separated into four parts.

In PLUSPULS as in most other TMS stimulators a capacitance *C* is used as an energy storage (Fig. 2.A). *C* is charged prior to pulsing to the voltage *V* by a high voltage power supply *V* and the maximum current of the stimulation pulse $I_{\max}$ is reached when $V(t_{\mathrm{Imax}})=0$. In the lossless case $I_{\max}$ is

(1) $$I_{\max}=V\sqrt{\frac{C}{L}}\text{.}$$

In practice, ohmic losses of the wire, the coil, and the capacitor as well as the losses at the switch must be considered as well. PLUSPULS is a biphasic TMS device. Biphasic TMS have the advantage that the capacitor recharges with the stimuli pulse (Fig. 2 B (blue)), thereby enabling fast pulsing. The energy and time needed to recharge the capacitor to $V_{C0}$ again is small, because only the losses during the stimuli have to be compensated. In PLUSPULS the pulse capacitor voltage after a stimulation pulse is around 80% of the voltage before to the stimulation pulse (Fig. 8).

In biphasic TMS setups, the TMS coil conducts a sinusoidal current pulse for the whole pulse duration (Fig. 2B) in the lossless case. The direction of the induced electric field changes with the sign change of the current slope. Each of the four pulse slopes in Fig. 2B part ① - ④ generates a similar electric field strength amplitude diminishing over time. The negative current during part ③ and part ④ recharges the capacitor before it is switched off.

The active phase of a biphasic pulse (Fig. 2B ② and ③), is the longest time with one current slope direction, hence, one *E* direction. This causes the highest voltage at the neuron membrane $V_{m}$ because of the charge accumulation at the neuron membrane over twice the duration compared to part ① which generates a higher electric field amplitude.

With the ohmic losses neglected, the voltage at *C* would be the same as before the pulse. The maximum current $I_{\max}$ is calculated with equ. 1 and the current $I_{\mathrm{bi}}$ over the entire period with equ. 2. The voltage $V_{\mathrm{bi}}$ can be described with equ. 3.

(2) $$I_{\mathrm{bi}}=I_{\max}\sin\left(\frac{1}{\sqrt{\mathrm{LC}}}t\right)$$

(3) $$V_{\mathrm{bi}}=V\cos\left(\frac{1}{\sqrt{\mathrm{LC}}}t\right)$$

Other stimulator types like the monophasic are simple but have a slower repetition rate because of the total discharge of the pulse capacitor [1]. Multilevel converters and similar designs provide customized pulse shape but the power electronics needed for this devices is more complicated than a biphasic stimulator [32], [33], [37], [38].

### 1.3 Pulse Protocols

**Fig. 3:**
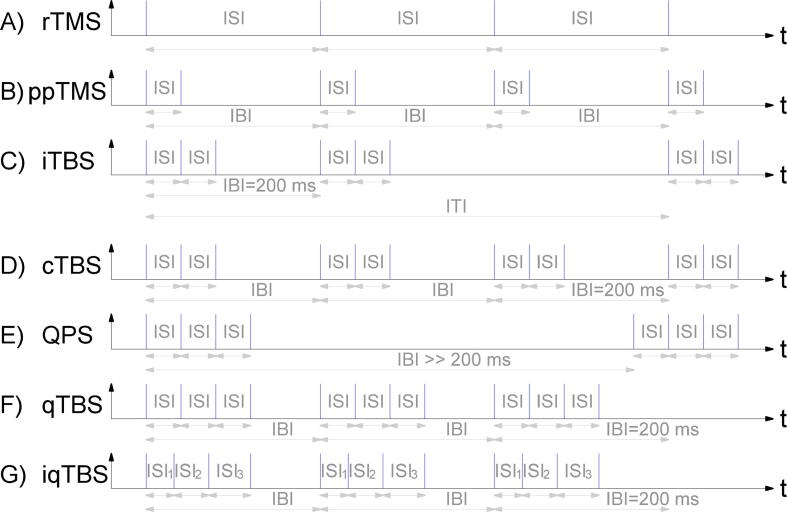
Pulse timing for different TMS pulse protocols. The stimulation pulses are illustrated as the blue vertical lines. The inter stimulus interval (ISI) describes the time delay between stimuli, the inter burst interval (IBI) the time delay between the starts of the bursts and the inter train interval (ITI) the time delay between starts of pulse protocols. The pulse protocols are (A) Repetitive TMS (rTMS), (B) Paired pulse TMS (ppTMS) (Fig. 4), (C) Intermittent theta burst stimulation (iTBS), (D) Continuous TBS (cTBS), (E) Quadri pulse stimulation (QPS), (F) quadri TBS (qTBS) combines TBS and QPS and (G) Individual qTBS.

Pulse protocols define the timing of stimulation pulses (Fig. 3). The three important parameters are the inter-stimulus interval (ISI) which describes the time between stimulation pulses, the inter-burst interval (IBI) defining the time between the first pulse of the bursts, and the inter-train interval (ITI) which is the time between the first pulse of a trains of bursts.

Single pulse TMS applies only one pulse to the brain region. It is often used as diagnostic tool [3], [4]. Single pulse TMS can for example applied to test the integrity of a motor-cortical brain circuit by pulsing the region and looking at the reaction of the peripheral muscle.

**Fig. 4:**
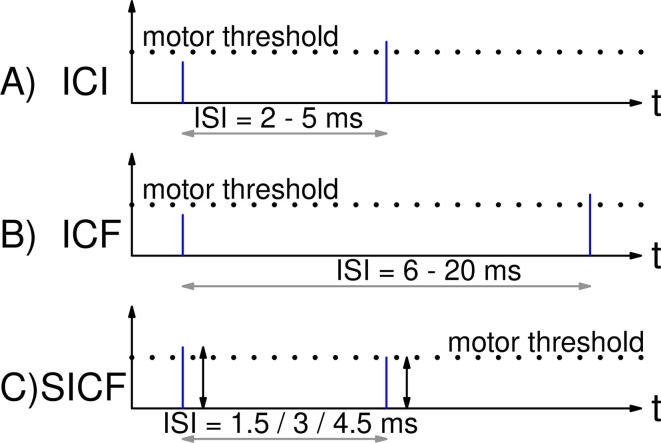
ppTMS for evaluating different physiological parameters. (A) Intracortical inhibition (ICI), a subthreshold conditioning stimuli with a supra threshold test stimuli with a ISI in between 25ms. (B) Intracortical facilitation (ICF) is the aforementioned stimuli configuration but with an ISI of 620ms. (C) The short latency intracortical facilitation (SICF) can be detected at specific ISIs around 1.5ms, 3ms and 4.5ms with supra- or perithreshold conditioning stimuli and with varying test stimuli amplitude.

There are several common pulse protocols available. The most common are repetitive TMS (rTMS) (Fig. 3 A), ppTMS (Fig. 3 B, Fig. 4), theta burst stimulation (TBS) (Fig. 3 C, D), quadri pulse stimulation (QPS) (Fig. 3 E) and quadri theta burst stimulation (qTBS) (Fig. 3 F). The different pulse protocols are challenging the hardware and software of the PLUSPULS in different ways and are used for the validation and characterization.

*repetitive TMS (rTMS)*. rTMS is the stimulation of brain regions with a fixed repetitive rate of pulses stated as frequency *f* (Fig. 3 A). This is in contrast to single pulse and ppTMS, which apply only one or two pulses. According to the frequency, low frequency rTMS f< 1Hz and high frequency rTMS f>1Hz are distinguished [4]. The focus of rTMS is on treatment. For example the approved TMS method for treating depression is based on a high frequency rTMS protocol [7].

Compared to a rTMS protocol, a rTMS burst protocol has no fixed repetition rate, but a short ISI between a small number of pulses, so called bursts, and high IBI between between the bursts.

rTMS burst protocols are frequently applied to modulate the cortico-spinal excitability, commonly referred to as long-term potentiation (LTP)-like and long-term depression (LTD)-like effects in human motor cortex (M1) [20], [26], [23]. The following rTMS burst protocols visualized in Fig. 3 are theta burst stimulation (TBS), quadripulse stimulation (QPS), quadri theta burst stimulation (qTBS), and individual quadri theta burst stimulation (iqTBS).

*Paired Pulse (ppTMS) Protocol*. The paired pulse (ppTMS) protocol applies two stimulation pulses with a small ISI to one brain region (Fig. 3 B). TMS using ppTMS is suitable for treatment and diagnosis.

It is used to determine or induce intracortical inhibition (ICI) (Fig. 4 A) or intracortical facilitation (ICF) (Fig. 4 B) of a brain region [4], [39]. It changes for different ISI, waveform [40], with different levels of the first stimuli, the conditioning stimuli [41], and the second stimuli the test stimuli, between intracortical inhibition and facilitation.

ICI can be detected with a subthreshold conditioning stimuli and a suprathreshold test stimuli at ISI in between 25ms [41] (Fig. 4 A). This is often called short latency intracortical inhibition (SICI). The highest inhibition level can be achieved with a subthreshold conditioning stimuli at 80 % of the motor threshold [41]. The conditioning stimuli has a significant influence on inhibition at and above a level of 70% of the motor threshold [42]. Rothwell et. al. [43] stated the maximum of inhibition with 90% of motor threshold for the conditioning stimuli.

ICF can be detected with a subthreshold conditioning stimuli and a suprathreshold test stimuli at ISI in between 620ms [44], [4], or 830ms [41] (Fig. 4 B).

Short latency intracortical facilitation (SICF) can be achieved with a suprathreshold conditioning stimuli and a subthreshold test stimuli [42], or two perithreshold stimuli [45] or two suprathreshold stimuli [46] with a specific ISI of around 1.5ms, 3ms and 4.5ms (Fig. 4 C). The influence of conditioning stimuli amplitude over different ISIs was researched [42]. Repetitive stimulation with subthreshold stimuli with the same amplitude for conditioning and test stimuli and an ISI of 1.5ms can induce LTP [47].

*Theta Burst Stimulation.* Theta burst stimulation (TBS) is a safe, consistent, and rapid method for inducing brain plasticity for longer than 60min after stimulation [48]. A theta burst consists of three pulses with ISI in the range of 20ms (50Hz) and an inter burst interval (IBI) of around 200ms (5Hz). It is often divided in between continuous TBS (cTBS) (Fig. 3 D) and intermittent TBS (iTBS) (Fig. 3 C). iTBS has a pause between a train of bursts and cTBS has a continuous stimulation with specified ISI and IBI [48]. The inter train interval (ITI) defines the time between the trains (Fig. 3). iTBS inserts a pause of 8s after a stimulation period of 2s. iTBS increases and cTBS decreases the cortical excitability[49].

*Quadri pulse Stimulation.* The quadri pulse stimulation (QPS) protocol is a burst of four stimuli with an ISI in a range of 1.5 - 100ms (Fig. 3 E). The IBI is set to 5s and the stimuli are applied with 90% of the (active) motor threshold [26]. Dependent on the ISI QPS can originate LTP or LTD [26], [49]. This protocol is more effective for plasticity induction than ppTMS stimulation [27]. A QPS protocol is often applied via four combined monophasic stimulators. Some studies show that a monophasic TMS is more efficient in plasticity induction [50] than a biphasic TMS but it is effective and allows to combine QPS with TBS to further increase efficiency [20].

*Quadri Theta Burst Stimulation*. Quadri theta burst stimulation (qTBS) combines the two previously mentioned protocols TBS with QPS (Fig. 3 F). Only one ultra high frequency biphasic stimulator is needed to generate pulses with this pulse protocol [28], [20]. The stimuli are applied with 90% of the motor threshold the same as for QPS[20]. The four stimuli per burst have an ISI of 1.5ms - 50ms similar to QPS and the IBI is the same as in theta burst stimulation with 200ms. qTBS with an ISI of 1.5ms or 5ms can induce lasting changes in cortico-spinal excitability [20].

*Individual Quadri Theta Burst Stimulation.* Individual quadri theta burst stimulation (iqTBS) derived from qTBS and separately adjusts the ISI times between the four biphasic stimuli per burst ${\mathrm{ISI}}_{1},{\mathrm{ISI}}_{2}$ and ${\mathrm{ISI}}_{3}$ (Fig. 3 G). The ISIs typically range between 150ms. Through individualized protocols, iqTBS can be a tool for tailored treatments [51]. The ISI can be adjusted to individual parameters of each patient like the SICF determined by a paired pulse sequence [46].

## 2 Hardware Description

**Fig. 5:**
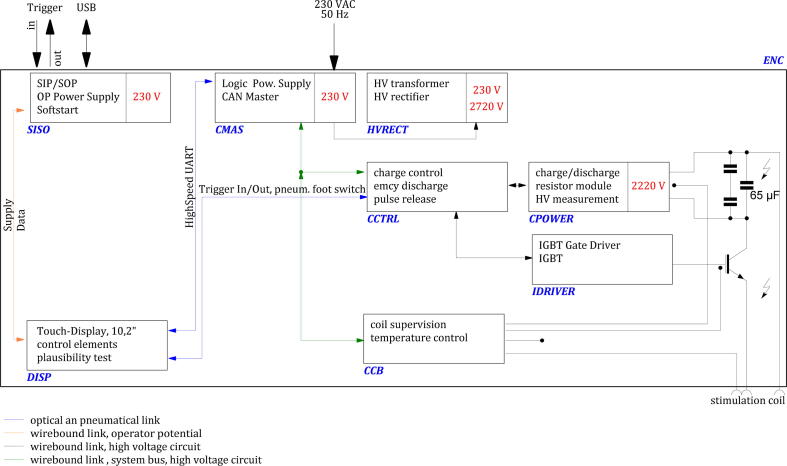
Overview of PLUSPULS TMS with a focus on the PCBs and the connection between each of them.

PLUSPULS consists of five micro controller (μC) with their printed circuit boards (PCB), three PCBs without μC, and many components not on PCBs (Fig. 5). The PLUSPULS is supplied with 230$V_{\mathrm{AC}}$, generates a 2720$V_{\mathrm{DC}}$ for the high voltage power supply and charges the pulse capacitor with a maximum of 2220$V_{\mathrm{DC}}$. The high currents of up to 4000A suggests that the distances between parts which conduct these currents are as short as possible to minimize losses and electromagnetic interference. These lethal voltages and the high currents require a high safety standard for the PLUSPULS.

The communication between PC and PLUSPULS is via UART on USB. The communication between CMAS, CCB and CCTRL is realized via system bus with CAN because of the inherent safety features (Fig. 5 green lines). The other communication interfaces are realized as UART because of the simplicity and the ability to interact via fiber optics.

*System Input System Output board*. The system input system output (SISO) board with its μC is responsible for the BNC input and output connections, the USB connector to communicate with the graphical user interface (GUI) on PC, the connection to the power plug, the voltage monitoring of the power supply, and the softstart of the high voltage power transformer (Fig. 5). The SISO is split into two parts with different ground potential. One part is on the housing potential and the second is on neutral or phase potential. The SISO supplies the DISP board with power and is connected with it via UART. The fuse of the high voltage transformer, the soft start, and the switch of the high voltage power transformer are also realized on SISO.

*DISPlay board*. The DISPlay board (DISP) is on the housing potential and is the interface between the UART connection to PC with the graphical user interface (GUI) for the PLUSPULS control, the UART connection to SISO and the UART via fibre connection to CAN MASter board (CMAS). It has two fibre connections to Charge ConTRL board (CCTRL) in order to trigger stimulation pulses and get feedback. It is the hub for the information via UART and is the main storage of the configuration data defined with the GUI.

CMAS supplies the other PCBs on the same potential with 24V and 5V. Its ground is on HV ground. It is also the interface between the CAN bus connection on HV ground and the UART connection via fiber optics to the DISP board on housing ground.

*Charge ConTRoL board*. CCTRL connects to the CMAS and Coil Control board (CCB) (Fig. 5) via system bus and is directly connected to IGBT DRIVER board (IDRIVER) and the Charge POWER board (CPOWER). The CCTRL board supplies the IDRIVER board with a positive and negative voltage via DC/DC converter to ensure a safe switch on and off of the IGBT and connects to IDRIVER via optocouplers. The emergency relay to discharge the pulse capacitor is placed here and needs also a separate potential. The pneumatic foot switch, to control operator presence, sends its signal to CCTRL. At each system start up the self test calibrates each of the four analog circuits of the high voltage measurements on CPOWER. Additionally the asymmetry detection realized in hardware is tested for different error cases. The tasks for CCTRL are the instruction to IDRIVER for the ignition of the stimulation pulse and the charge control via the charge IGBTs on the CPOWER board.

*IGBT DRIVER board*. IDRIVER is a small PCB mounted directly on the gate of the pulse IGBT. Its ground is connected to the emitter of the IGBT, which has a different potential than HV ground. The separation of the voltage potentials is realized on CCTRL. IDRIVER drives the gate and provides feedback on the state of the IGBT. A two-stage driver built and a dual power supply with low resistive capacitors ensures a fast switch on and off time of the gate.

*Charge POWER board*. CPOWER is the board to control the current flow to and from the pulse capacitor. Its IGBTs on different potentials than HV ground are supplied with positive and negative voltage via DC/DC converters to ensure a safe switch on and off and are connected to the board via optocouplers. The conversion of the high voltage at pulse capacitor and high voltage power supply to measurable values via resistive voltage divider and the analog circuit is realized on the CPOWER board. The CPOWER board is the only board with six layers. The main part of the CPOWER board is on HV ground potential which is located in the middle of the 2720V of the high voltage power supply. The absolute number of the voltage measured to the positive and negative side should be the same if everything is fine. An asymmetry can be caused by errors in the pulse capacitor, the high voltage power supply capacitors or in the charging circuit and would lead to a change of the rated voltage on which the calculation of the clearance and creepage distance is based, which leads to insufficient insulation between HV ground and other potentials (Table 2). In case of error we differ between a static and dynamic error. They have different voltage limits and timing constraints.3.

**Table 2:** Insulation distances for creepage and clearance based on the Tables 12, 13, 14, 15 and 16 in standard DIN EN 60601–1:2013 [52], table A.2 from standard IEC 60664–1:2002 [53], and Table 4 and 6 from standard EN 50178:1997 [54].

No	Safety	rated Voltage	test voltage	Clearance/mm	Creepage/mm
1	1xMOOP	230$V_{\mathrm{AC}}$	-	2.0 (Table 13)	2.3 (Table 16)
2	2xMOOP	230$V_{\mathrm{AC}}$	1500$V_{\mathrm{AC}}$	2.0 (Table 13)	2.3 (Table 16)
3	1xMOOP	2220$V_{\mathrm{DC}}$	2815$V_{\mathrm{AC}}$	8.4 (Table 15)	22.1 (Tab. A.2)
4	2xMOOP	1214$V_{\mathrm{AC}}$=1720$V_{\mathrm{DC}}$	3257$V_{\mathrm{AC}}$	12 (Table 13 + 14)	23.8 (Tab. A.2)
5	2xMOOP	960$V_{\mathrm{AC}}$=1360$V_{\mathrm{DC}}$	2767$V_{\mathrm{AC}}$	4.2 (Table 15)	13.6 (Tab. A.2)
6	2xMOPP	230$V_{\mathrm{AC}}$	4000$V_{\mathrm{AC}}$	5.0 (Table 12)	8.0 (Table 12)
7	2xMOPP	2220$V_{\mathrm{DC}}$	8125$V_{\mathrm{AC}}$	28.6 (Table 12)	48.7 (Table 12)
8	2xMOPP	230$V_{\mathrm{AC}}$	4000$V_{\mathrm{AC}}$	5.0 (Table 12)	8.0 (Table 12)
9	2xMOPP	230$V_{\mathrm{AC}}$	4000$V_{\mathrm{AC}}$	5.0 (Table 12)	8.0 (Table 12)
10	2xMOOP	2150$V_{\mathrm{AC}}$ = 3040$V_{\mathrm{DC}}$	4735$V_{\mathrm{AC}}$	13.7 (Table 13 + 14)	30.4 (Tab. A.2)
11	only functional	1360$V_{\mathrm{DC}}$	1360$V_{\mathrm{DC}}$	5.3 (Table 4)	6.8 (Table 6)
12	2xMOOP	2720$V_{\mathrm{DC}}$	4147$V_{\mathrm{AC}}$	8.4 (Table 15)	27.2 (Tab. A.2)
13	2xMOOP	1920$V_{\mathrm{AC}}$ = 2720$V_{\mathrm{DC}}$	4000$V_{\mathrm{AC}}$	8.4 (Table 15)	54.4 (Tab. A.2)
14	only functional	2220$V_{\mathrm{DC}}$	2220$V_{\mathrm{DC}}$	9.0 (Table 4)	11.1 (Table 6)
15	2xMOOP	230$V_{\mathrm{AC}}$	3000$V_{\mathrm{AC}}$	4.0 (Table 13)	4.6 (Table 14)

**Table 3:** Connector components for the socket mounted to the housing.

Description	Details	Part number	Vendor	Order Number	Quantity
Bottom part, panel montage	Han Eco	1941 006 0301	Farnell	1892236	1
Female screw contacts	Han Modular	0914 002 2741	Farnell	2842857	2
Female contact module, 12 pin	Han Modular DD	0914 012 3101	Farnell	728147	1
Female contacts, AWG 20	Han D	0915 000 6203	Farnell	659370	10

*Coil Control Board*. CCB is connected to the temperature sensors in the stimulation coil and most of CCB is on floating potential. It measures the stimulation coil temperature and the correct connection to the stimulation coil. CCB is connected via DC/DC converter and optocouplers to the connectors on HV ground on its board. At system start the correct connection to the stimulation coil, analog circuits for the temperature measurement and error detection are tested.

*High Voltage RECTifier board*. The high voltage rectifier board (HVRECT) serves as a placement of the rectifier and the energy storage capacitors of the high voltage power supply. The secondary side of the high voltage power transformer is the input of HVRECT and the outputs are the positive high voltage supply, the negative high voltage supply and the HV ground in the middle of both. The negative high voltage supply will be connected to the housing.

The high voltage generation with a toroidal transformer is simple, low component numbers and is low on electro magnetic interferences (EMI) which allows compatibility with electroencephalography devices.

*IGBT*. The IGBT FZ1000R33 is switched on to start the stimulation pulse (Fig. 2 B). The IGBT is switched off during the second half of pulse when the internal diode conducts the current (Fig. 2 B ③ and ④). The following change in current polarisation switches off the diode. It is conducting some limited time although in opposite direction. This effect is called reverse recovery effect and leads to current flow although the IGBT module is completely switched off. Switching off this current through the inductance of the stimulation coil causes an over-voltage. In order to limit this over-voltage a snubber is placed in parallel to the IGBT. The snubber consists off a 6.8Ω resistor and a 2μF capacitor. This design can be improved by shortening the cables between snubber and IGBT, and by replacing the wire-wound resistor with a resistor with low inductance (Fig. 33). The snubber was sufficient to limit the over-voltage to values lower than the voltage withstanding capability of the FZ1000R33 (3300V). An IGBT was preferred as switch (Fig. 2 A). A thyristor would need a pulse duration longer than twice its reverse recovery time. The targeted minimum pulse duration is 160ms. This is challenging for thyristors.

*Pulse Capacitor*. The capacitance of the pulse capacitor is 65μF. Combined with an maximum voltage of 2220V the energy stored equals 160J. This is the same as https://magandmore.com/de/produkte/produkte-forschung/the PowerMAG series of MAG & more so the maximum stimulator output (MSO) is comparable. The TMS [28] developed for Quadri-Pulse Theta Burst Stimulation [20] has the same capacitance of 65μF but a higher maximum voltage of 2700V.

**Fig. 6:**
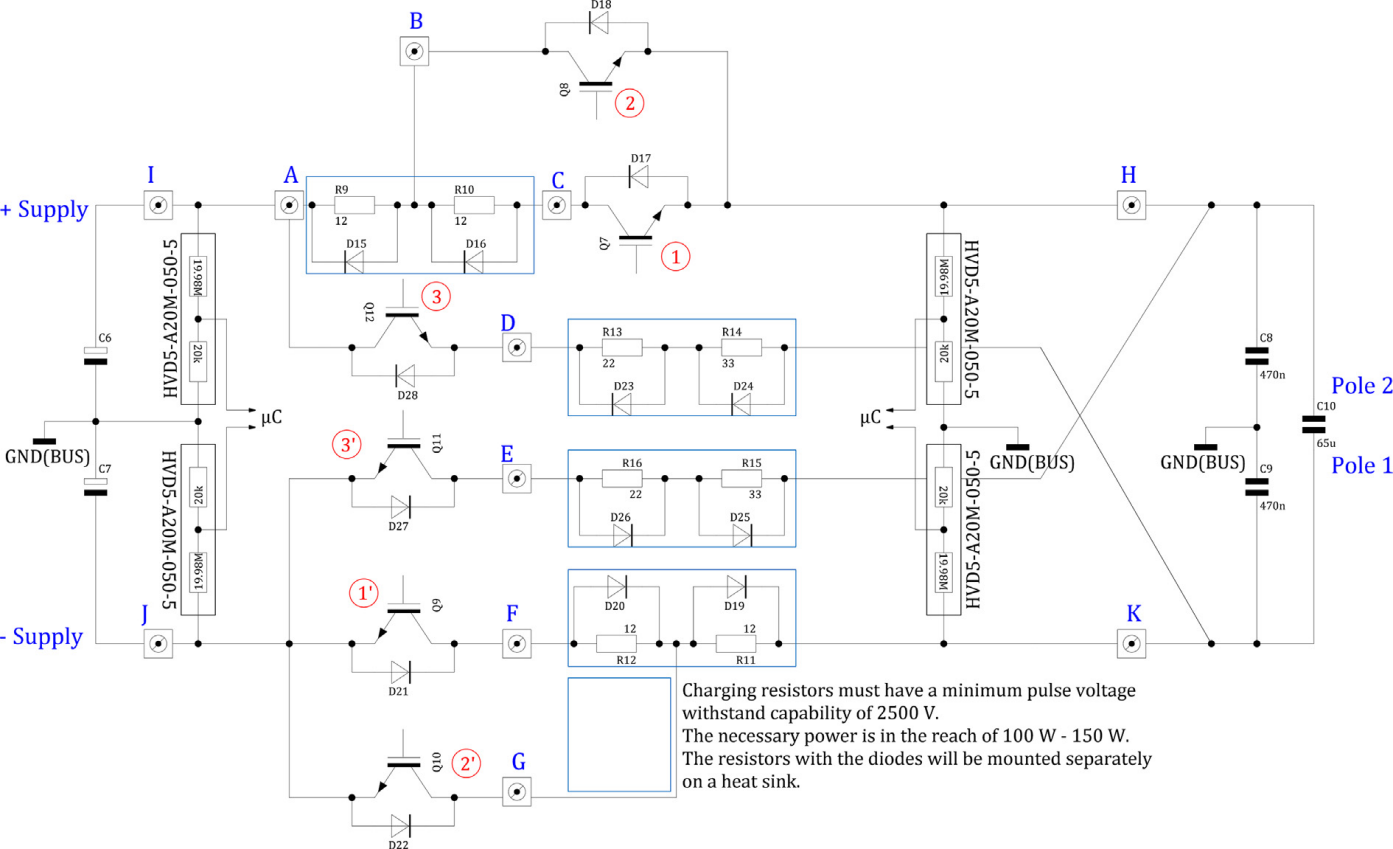
Overview of high voltage power supply and the charging network for the pulse capacitor with the two capacitors for the stabilization of the voltage at half of the high voltage power supply. The letters mark the RED Cube connectors on the CPOWER board.

*Charging Network*. The charging network is between the high voltage power supply and the pulse capacitor (Fig. 6). Each charging mode, slow charge (Fig. 6①, ①’), fast charge (Fig. 6 ②, ②’) and discharge (Fig. 6 ③, ③’) needs one IGBT for each voltage side resulting in six IGBTs needed. The resistors are mounted on a cooling rack and a parallel diode reduces possible over-voltage at switch off on the wire-wound resistors. The current flow during fast charge mode is around double the amount during slow mode, because the resistance in the charging circuit is halved. The resistance of the discharge network is higher in order to restrict the maximum current flow in each path to comparable levels. During discharge the maximum voltage difference is the maximum high voltage supply voltage (2720V) plus the maximum voltage at the pulse capacitor (2220V) resulting in 4940V compared to the 2720V during slow and fast charge.

### 2.1 Hardware Description Summary

• *PCBs with dedicated functions*
• *modular system*
• *different PCB ground potentials*
• *high voltage*
• *high current*

## 3 Design Files Summary

.

**Table:** 

**Design filename**	**File type**	**Open source license**	**Location of the file**
CCB	Altium Project	CC 4.0 International	doi.org/10.5281/zenodo.6457647
CCTRL	Altium Project	CC 4.0 International	doi.org/10.5281/zenodo.6457647
CMAS	Altium Project	CC 4.0 International	doi.org/10.5281/zenodo.6457647
CPOWER	Altium Project	CC 4.0 International	doi.org/10.5281/zenodo.6457647
DISP	Altium Project	CC 4.0 International	doi.org/10.5281/zenodo.6457647
HVRECT	Altium Project	CC 4.0 International	doi.org/10.5281/zenodo.6457647
IDRIVE	Altium Project	CC 4.0 International	doi.org/10.5281/zenodo.6457647
SISO	Altium Project	CC 4.0 International	doi.org/10.5281/zenodo.6457647
CREPEXT	CAD file	CC 4.0 International	doi.org/10.5281/zenodo.6457647
GUI	Matlab GUI	CC 4.0 International	doi.org/10.5281/zenodo.6457647
common	C library	CC 4.0 International	doi.org/10.5281/zenodo.6457647
stm32f4	C library	CC 4.0 International	doi.org/10.5281/zenodo.6457647
CCB	Keil project	CC 4.0 International	doi.org/10.5281/zenodo.6457647
CCTRL	Keil project	CC 4.0 International	doi.org/10.5281/zenodo.6457647
CMAS	Keil project	CC 4.0 International	doi.org/10.5281/zenodo.6457647
DISP	Keil project	CC 4.0 International	doi.org/10.5281/zenodo.6457647
SISO	Keil project	CC 4.0 International	doi.org/10.5281/zenodo.6457647

An Altium project (.prjpcb) incorporates the schematics (.schdoc) for the PCB design (.pcbdoc), the manufacturer rules, and the bill of materials (.xls) of the PCB. A Keil project (.uvprojx) is the C Code (.c +.h) for the STM32 μC on the corresponding board including the necessary libraries and development tools.

The μC software can be found in the package pcb_software and the altium projects in the Hardware1 and 2 package.

The following PCBs in the itemized list can be seen in Fig. 5.

• CCB is the coil control board, its PCB and software.
• CCTRL is the charge control board, its PCB and software.
• CMAS is the CAN master board, its PCB and software.
• DISP is the display board, its PCB and software.
• SISO is the system input system output board, its PCB and software.
• CPOWER is the charge power board pcb design.
• HVRECT is the high voltage rectifier board pcb design.
• IDRIVE is the IGBT driver board pcb design.
• HMI is the folder of the Matlab Code for the GUI. The GUI can be opened by the Home.mlapp file.
• CREPEXT is the 3D-printed creepage extension needed for the HAN ECO plug to fulfill the requirements.
• common is the first selfmade C library included in other software projects.
• stm32f4 is the second selfmade C library included in other software projects.

## 4 Bill of Materials Summary

Table 1 summarizes the expensive PLUSPULS parts. In most cases they are not part of a PCB. The bill of materials (BOM) of each PCB is part of the corresponding Altium project folder in repository files Hardware1 and 2. The cables and their length depend on the built up.

**Table 1:** Main cost units of the electrical hardware.

Description	Part Number	MFR	Vendor	Order Number	No	€/Unit
IGBT	FZ1000R33	Infineon	Digikey	FZ1000R33HL3BPSA1-ND	1	1,728
pulse capacitor	ER30-256	Vishay	-	-	1	-
pulse capacitor	ER30-206	Vishay	-	-	2	-
transformer	1660 VA (customized)	Induktor	-	-	1	300
supply capacitor	B43630A5108M000	EPCOS	Mouser	871-B43630A5108M000	16	13.22
charge resistors	HS100/150	ARCOL	-	-	9	10
resistor dividors	HVD5-A20M-050–05	Caddock	Mouser	684-HVD5-A20M	4	41.77
IGBT	IXEL40N400	IXYS	Mouser	747-IXEL40N400	6	106.89
coil plug	HAN Eco	Harting	-	-	1	143
coil socket	HAN Eco	Harting	-	-	1	194
connector	7464000	WE	Mouser	710–7464000	13	8.51

## 5 Build instructions

The high voltage in PLUSPULS requires safety. The description of the required safety and the safety features incorporated in the PLUSPULS should be the foundation to built the PLUSPULS. In the appendix are the photos of the PCBs, subsystems and the PLUSPULS that allow to rebuilt PLUSPULS with the help of the schematics in the repository and the figure captions which explains the interconnections.

### 5.1 Safety

PLUSPULS is a medical device operating with voltages of up to 2720V and currents up to 4000A. Commissioning this device is only allowed for an electrician. Crafting this device does not need the same skill level. We advice testing the device with two people so in case of an accident, the second can help. All legal requirements of the country for the handling of high voltages have to be applied.

The design for a medical device requires first error security. This can be addressed by special safety features in hardware and/or increased creepage and clearance distances. Stimulating probands or patients requires the operator to know the risk involved, such as seizure induction or the heating of metallic implants [51].

#### 5.1.1 Safety Features

Each safety relevant board (CMAS, CCTRL, CCB), which are connected via the system bus (Fig. 5 green line), incorporates two hardware safety features. The first is an internal control of safety relevant signals on the board combined with a high signal which verifies the safe start of the μC. Additionally a toggling output of the μC indicating operability (software watchdog), which is converted to a high signal by monostable flip-flop, if the μC is running (hardware watchdog). On every board the voltage control of every supply voltage is safety relevant. Other signals like the voltage control of the pulse capacitor are board specific and are incorporated into the safety logic on this specific board. The output signal of this safety logic is called BOARD OK. The output of the μC signalling a safe start can be used to test the safety logic by toggling the output and supervising the BOARD OK.

The second safety feature is the emergency stop loop (E-STOP). The E-STOP has a high signal if every BOARD OK has a high signal. CCTRL, CCB and CMAS are connected via two optocouplers to the E-STOP. The first optocoupler transmits the BOARD OK signal to the loop. The second transmits the E-STOP signal to each board. The loop consists of one current source in series with the transistors controlled by the first optocoupler and the LEDs of the second optocouplers. This design is robust against electromagnetic interference. If an error occurs on one board, the BOARD OK of this PCB should go low and hence the E-STOP should go low. Both signals are connected to each μC via an interrupt which starts an emergency thread and an emergency message.

The E-STOP has to be high to change PLUSPULS from the PREOP into the OP mode and to stay there.

A test at each startup is done to ensure the safety features of the boards. Toggling μC outputs to test the safety logic or applying an analog voltage to a circuit to calculate and calibrate the amplification and offset is just one part of it.

#### 5.1.2 Voltage Calculation for Creepage and Clearance distance

The high voltage for the pulse capacitor is generated by a transformer with two 960V secondary voltage outputs. Rectifying both outputs in series results in $2\;*\;960V_{\mathrm{AC}}*\;\sqrt{2}=2715V_{\mathrm{DC}}$ for the high voltage power supply. Rounding it up to 2720$V_{\mathrm{DC}}$ and locating the ground of CMAS, CCTRL, CCB and CPOWER on HV ground results to 1360$V_{\mathrm{DC}}$. The charging circuit ensures that the maximum voltage at the pulse capacitor does not exceed 2220$V_{\mathrm{DC}}$. The device is developed for the European market and the network voltage is assumed to be 230$V_{\mathrm{AC}}$ pm 10% with 50Hz.

PLUSPULS operates with voltages up to 2720V and therefore, high safety requirements, especially as a medical device, are necessitated. Special safety is required regarding the patient and proband. The standard IEC 60601–1 [52] differentiates between operator protection (OP) and patient protection (PP), as well as means of patient protection (MOPP) and means of operator protection (MOOP). For the specific required insulation, the maximum possible voltage between two places has to be calculated. It has to be checked if one of these can be touched by the operator or the patient.

For this device, there are some assumptions and restrictions. First, PLUSPULS is only allowed to operate on altitudes lower than 2000m. In this case, we do not need factors to increase the clearance.

Second, the degree of pollution is assumed to be 2 as used in [52] table 15, because the housing has to be slit to enable the air cooling of the charging resistors which enables dust and humidity to enter. The material group is assumed to be IIIb for FR4, the base material of the PCBs, as used in [52] table 16. The only active component in contact with the patient is the coil, but it is not part of PLUSPULS.

The creepage (Table 2) is calculated with the assumption, that the degree of pollution is 2. The values for the creepage and clearance were calculated based on the voltage and the tables in the standard. Which table has to be considered is mentioned in the bracket. Insulation, in regards to the housing, can be done with a simple MOP instead of the double because of the low resistance of the connection between housing and protective earth which already fulfills one safety requirement. This is relevant for the insulation distances 2, 5 and 12. Therefore, the distances mentioned in Table 2 are already 1xMOOP.

The stimulation coil, as the active part in connection with the patient, is not part of PLUSPULS and needs a certificate for medical use with fitting max. ratings for voltage and current. For testing purposes, a self built coil completely insulated with acrylonitrile butadiene styrene is also used.

**Fig. 7:**
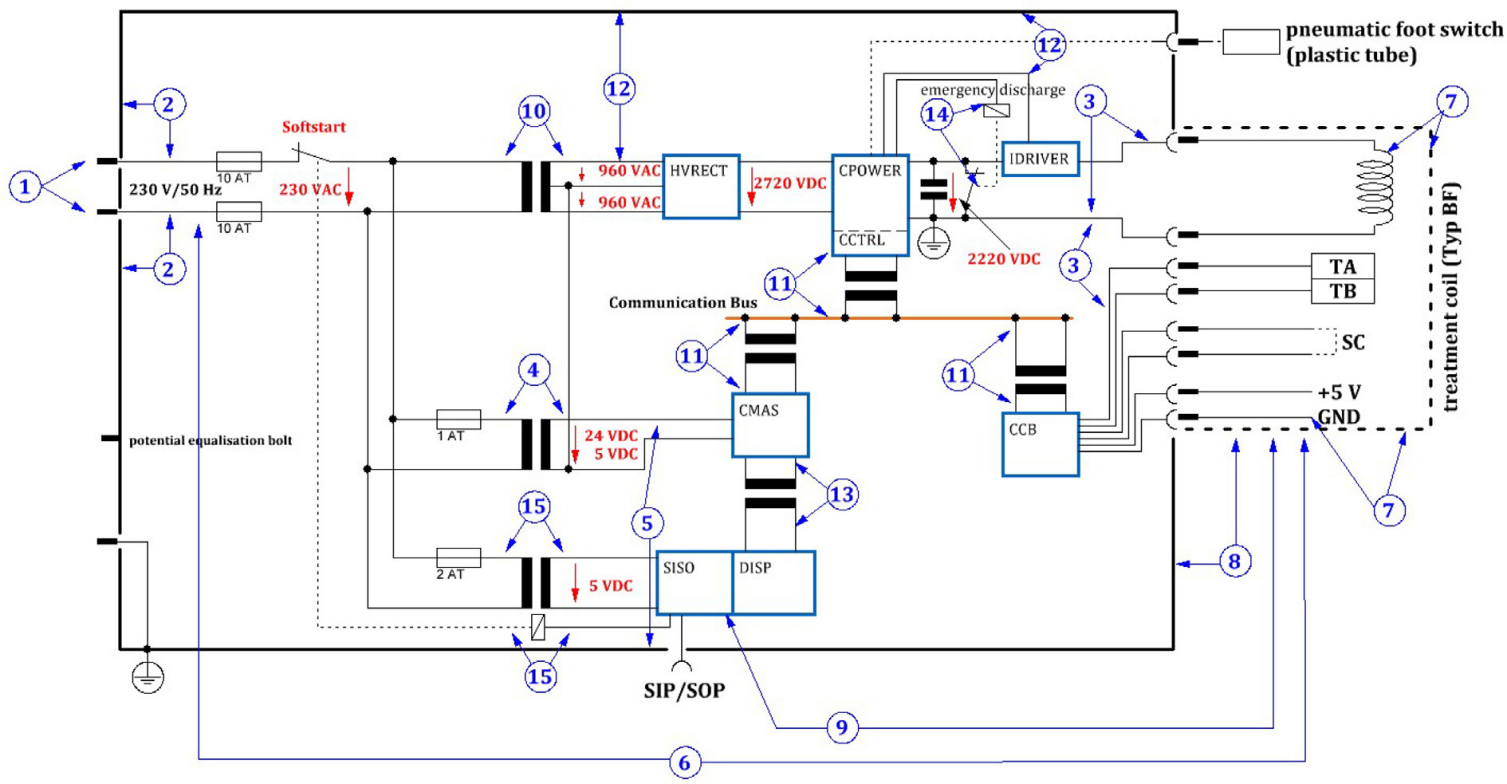
Overview of PLUSPULS with focus of the insulation between boards and housing.

The following enumeration describes the insulation distances, shown in Fig. 7 and stated in Table 2, sorted by its corresponding numbers. Table 12, 13, 14, 15, and 16 are from standard DIN EN 60601–1:2013 [52]. Table A.2 is from standard IEC 60664–1:2002 [53]. Table 4 and 6 are from standard EN 50178:1997 [54].

1. The isolation between phase and neutral is only relevant for the operator. The clearance is chosen according to [52] Table 13. The creepage is a result of interpolation between two values in [52] Table 16.
2. Same as 1: but with double the safety, because the housing can be touched by the operator. The housing is grounded so only to first MOOP is needed because of the first error security.
3. The clearance is chosen from [52] Table 15 for $V_{\mathrm{DC}}$ up to 2800V. The creepage is the result of interpolation from [53] Table A.2.
4. The ground of the secondary side of the high voltage transformer is connected to the center tap of the high voltage power transformer. The voltage is the sum of three voltages. The voltage at the center tap of the high voltage power transformer (960$V_{\mathrm{AC}}$), the voltage at the primary side of the high voltage transformer (230$V_{\mathrm{AC}}$) and the secondary side (24$V_{\mathrm{DC}}$). The clearance is found with the help of [52] Table 13  + 14 and the creepage with [53] Table A.2. For the transformers, the material group is assumed to be II.
5. The voltage is between HV ground and the housing. [52] Table 15, for clearance, and [53] Table A.2, for creepage, was used to determine the distances. Only 1xMOOP is relevant.
6. The patient protection at the stimulation coil requires an enhanced safety which results in a 2*MOPP. The net voltage of up to 250 V combined with Table 15 [52] results in these creepage and clearance distances.
7. This insulation distances are also calculated with [52] Table 12 and the max. high voltage at the coil of 2220V.
8. Same as 6.
9. Same as 6.
10. The voltage is the sum of both secondary voltages of the HV power transformer and the primary voltage. The creepage and clearance can be found in the same way as in insulation distance 4.
11. Only functional. Only realized between HV ground and CCB. The relevant Tables are 4 and 6 of [54].
12. The voltage is the max. DC voltage of the high voltage supply. Clearance from [52] Table 12, creepage from [53] Table A.2.
13. In comparison to 12, the second MOOP can not be realized with the grounded housing, so the creepage is the full 2*MOOP.
14. Only functional. The voltage is the max. voltage and the pulse capacitor as stated in insulation distance 7. The calculation is the same as in insulation distance 11.
15. Same as insulation distance 2 but not with a grounded housing, so both MOOPs apply.

**Table 4:** Connector components for the connector mounted to the coil plug.

Description	Details	Part number	Vendor	Order Number	Quantity
Coil plug housing (straight)	Han Eco	1941 106 0422	Farnell	2576134	1
Coil plug housing (angled, alt.)	Han Eco	1941 106 0522	Mouser	617–19411060522	1
Male screw contacts	Han Modular	09 14 002 2646	Farnell	2842847	2
Male contacts module, 12 pin	Han Modular DD	09 14 012 3001	Farnell	728135	1
Male contacts, AWG 22	Han D	0915 000 6104	Farnell	847495	10

**Fig. 8:**
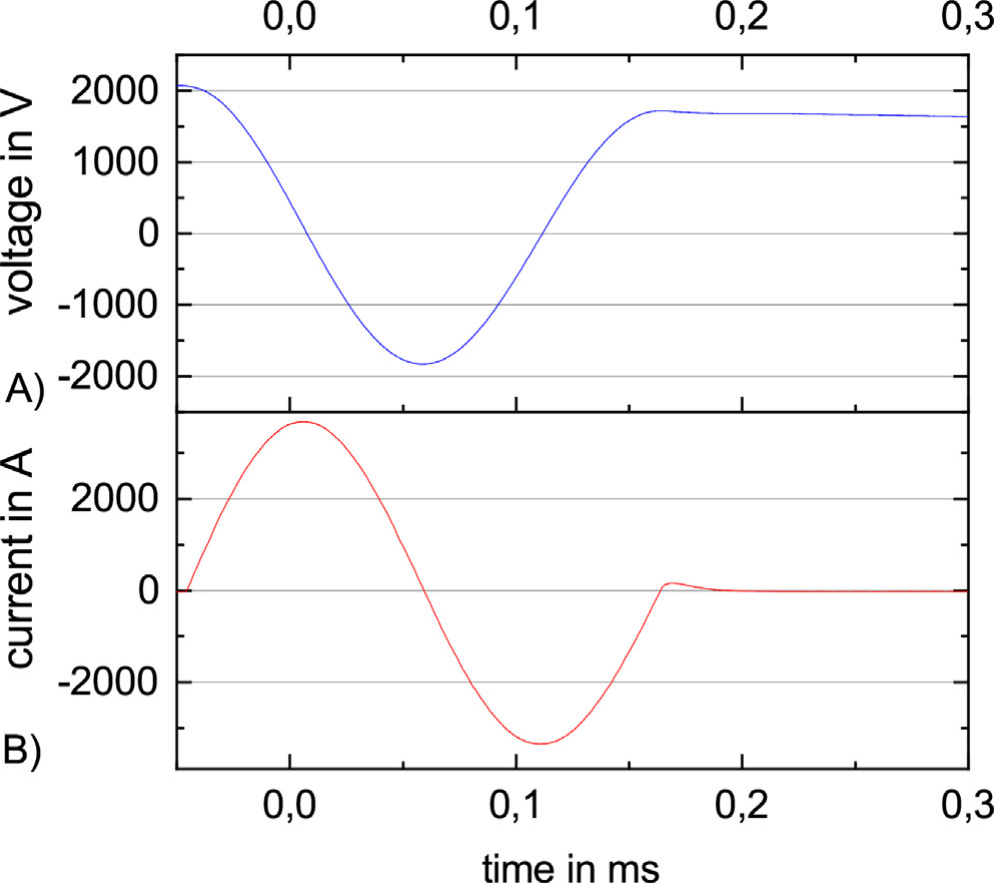
A single stimulation pulse with phases before the pulse, the pulse, a reaction time after the stimulation pulse and the discharge of the pulse capacitor. (A) The prior charged voltage at the pulse capacitor before the pulse, the damped cosine period during the pulse, the decreasing voltage during the return recovery current through the diode, no voltage change during the rest of the dead time and the decrease during the discharge after the stimulation pulse. Around 80% of the initial voltage is recovered after the pulse. (B) The current through the stimulation pulse with no current flow before the stimulation pulse, the damped sine wave during stimulation pulse, the reverse recovery current of the diode directly after pulse and the following idle.

#### 5.1.3 Creepage and Clearance on Printed Circuit Boards

The PCBs also have to apply the needed clearance and creepage distances. In most cases, the clearance is realized with the creepage distance length. In some cases, devices bridging these insulation like optocouplers or DC/DC converters have insufficient distance between pins on each side. A board cut out can elongate the creepage to a sufficient value. The width of the board cut out has to be more than 2mm to be regarded as insulation.

#### 5.1.4 Printed Circuit Boards

The PCBs ware designed in Altium Designer and based on this manufactured by Beta Layout. The part placement on the PCBs was handled by the author and was supported by a SMD insertion machine Expert-M from Essemtec AG. The reflow oven is a RO250 manufactured by Paggen GmbH. The solder paste placement was done with a pattern in a pattern holder. The thickness of the pattern is chosen in regards to the minimum pitch on the PCB. A low pitch requires a thin pattern. For the PCBs with μC, a thickness of 100μm was chosen.

Alternatively, some PCB manufacturer provide a placement service for an additional fee. The Bill of Materials (BOM) needs to be sent to the manufacturer and the left over parts will be returned with the populated board. In this case, the aforementioned tools are not needed. The μC on the PCBs were programmed via a 10 pin JTAG connector with the assistance of an ULINK PRO debugger from ARM Inc. DO NOT connect to the CMAS, CCB and CCTRL boards without disconnecting the high voltage power transformer FIRST.

#### 5.1.5 Housing

As part of the safety concept the housing has to be conductive and every part must be connected to the protective earth (PE) conductor from the low voltage system. The suggestion is a bolt working as a hub. The bolt is pressed into one of the housing parts which connects directly to every other housing part and the PE. The connection between bolt and part is realized with a copper cable with green/yellow color. The ends of the conductors are crimped with round terminals which are fastened to the bolt with a contact disk to the part, plains between round terminals and a spring washer at the nut. In the current breadboard state all PCBs are mounted on a wooden board for insulation, but the final state requires a noninflammable conductive housing.

If the insulation distances based on 12 (Fig. 7, Table 2) are underrun it is possible to elongate the distances by insulating the housing with polyimide tape with fitting test voltage withstand capability glued to the inside of the housing.

The main inlet needs 10A time-lag fuses for phase and neutral connection (Fig. 7). We used a Schurter KMF1.1163.11 with additional filtering properties fitting IEC 60939. The components are mounted on a wooden board for insulation. The housing is not realized yet. The housing has to be slotted to enable the cooling of the system. The SISO and DISP are meant to be mounted, with a spacer, on conductive parts of the housing. The SISO surrounds the main inlet and the holes in the housing for the two BNC and one USB connection have to be accurate. A push button to start a stimulation pulse, an inlet for the pneumatic foot switch, and the plug to connect to the stimulation coil need a cut out in the housing.

#### 5.1.6 Cable and Conductors

The high voltage cable should fit the maximum voltage of 2720V and the corresponding testing voltage of 4147V. We suggest the Silivolt-HV or Silivolt-2 V cables from Stäubli International AG. The suggested cables are litz wires. The litz wires after wire stripping increase their mechanical cross section and terminals with a higher cross section than the nominal cross section of the litz wire are recommended. As insulating hose we suggest the ISY series from Lapp with a min. thickness of 0.7mm or the ISS series from Lapp with a min. thickness of 0.5mm. As connection to the M8 screw terminals on the IGBT and the M6 on the pulse capacitor the terminals 01–0120102 and 01–0120101 from EAP GmbH with a maximum cross section of 10mm^2^ were crimped to the high voltage cable. The terminals were chosen to fit in the thick insulation of high voltage cables, with the drawback that the cross section of the terminal is oversized compared to the cross section of the cable.

The connection between the pulse capacitor, the pulse IGBT, and the connector for the stimulation coil should be as short as possible to minimize conductive losses and parasitic inductance. The connection between pulse capacitor and IGBT is realized with a 50mm^2^ copper bus bar.

A flat plug is used to connect the HV transformer on both primary and secondary side to the PCBs, the SISO to the main inlet, the primary side of the HV power transformer to SISO and CMAS with 1360V potential on HVRECT.

The system bus connections on HV ground between the boards are realized with cables fitting the AWG of the pins for the WR MPC3 connectors from Würth Elektronik GmbH. For best practice, the cables should be insulated by a insulation hose because of the 1360V between HV ground and housing. Otherwise, it must be ensured that the distance from the cable to other potentials with more than the required clearance distance defined in Fig. 7. For other connectors on the PCBs, the counterparts with the fitting cables have to be chosen.

The optical fiber cables in PLUSPULS are 1m long. The optical fiber is made from polyethylene material and 2.2mm in diameter with one HFBR 4503 and one HFBR 4513 connector on the end of the fibre and sold as 6112400100 by InSoft Uwe Flick. They match the HFBR or AFBR receivers and transmitters mounted on DISP, CMAS, and CCTRL.

#### 5.1.7 Stimulation Coil Connector

The connector to the stimulation coil has to meet the minimum required 22.1mm creepage and 8.4mm clearance distances (insulation distance 3 Table 2/ Fig. 7). The HAN ECO 6B from HARTING meets these requirements in the following build with an additional 3D printed insulation frame. The frame has to be mounted from the outside and put through the cutout in the housing for the connector. The screws to mount the connector and the frame to the housing are the same. The file can be found https://doi.org/10.5281/zenodo.6457647as CREPEXT in the Zenodo repository.

#### 5.1.8 Special Tools or Components

Allen Keys, screwdrivers, spanners, metric screws, and so on are considered normal tools available in a lab or workshop and are not mentioned here.

Soldering irons, for the through hole components on the PCBs, are needed because they have to be added after the reflow process in the oven.

Programming and debugging needs the aforementioned debuggers and development environment (Section 5.1.4).

It is advisable to use nylon screws for the fixation of the PCBs on HV ground. Nylon screws are non conductive and provide the needed clearance and creepage distance. Otherwise, the needed distances have to be provided from the socket and not from the PCB.

It is advisable to elongate underrun insulation distances between the housing and other potentials by gluing polyimide tape with fitting width and voltage withstand capability to the inside of the housing. The holes in CCTRL and CPOWER are meant to mount the CCTRL with spacers back to back to CPOWER, but the nylon screws are needed because of the different voltage potentials around the charge IGBTs.

Please note that the connectors listed in the BOM of a PCB also need counterparts for the cable.

Special crimping tools, mentioned in Section 5.1.6, are needed for the connectors.

An automated wire stripper is suggested to prepare the cables for crimping to the connector pins.

## 6 Operation Instructions

The operation of PLUSPULS is completely controlled from a GUI in Matlab. Other possibilities as a touch display or a spring return button at the front of the housing have interfaces and can be realized.

The GUI was developed with the help of the Matlab Design App. It is object oriented and consists of several.mlapp and.m files and needs the Matlab version 2020b or newer. The GUI is self explanatory and starts with the Home.mlapp file. An example is shown in the appendix.

After the successful PLUSPULS startup, the complete configuration, and a green signal in the top right of the GUI, the stimulation pulses can be generated by pressing and holding the button while standing on the pneumatic foot switch.

## 7 Validation and Characterization

PLUSPULS is made for high frequency stimulation of up to 1000Hz. The ISI was designed to be as low as 1ms. This ISI can’t be achieved continuously but for short bursts of stimuli of limited amplitudes. PLUSPULS has different limits for continuous stimulation and for short bursts. Based on this, three different sequence types were chosen to characterize PLUSPULS. All following sequences are configured in the Matlab GUI and programmed to PLUSPULS.

For the first one, we want to describe the maximum possible amplitude in steps of 10% MSO for a rTMS repetition rate in Hz. The % MSO amplitude is measured as the peak current through the stimulation coil.

The second is the paired pulse sequence (ppTMS) with an IBI of 1s. We describe three different cases of ppTMS. Pairs with equal stimulation output, pairs with a higher amplitude for the first stimulation pulse and pairs with a higher amplitude of the second pulse.

The third and last sequence in our characterization is the qTBS, a burst of four stimuli with a IBI of 200ms. For the definition of the % MSO over ISI, the three ISIs and their four stimuli amplitudes are the same. An example of a special individualized qTBS with a variation of ISIs and a second with an additional variation of the amplitudes illustrate the variability of PLUSPULS.

All acquisitions were done with a Rhode & Schwarz RTO2014 oscilloscope. Three probes are connected to the oscilloscope. A rogowski current transducer cwt 60b from Power Electronic Measurements with a divider ratio of 0.5mAV^−1^ to measure the current through the stimulation coil. A rogowski current transducer cwt 3b from Power Electronic Measurements with a divider ratio of 10m AV^−1^ to measure the charging current of the pulse capacitor. A differential high voltage probe SI-9010A from Sapphire Instruments with a divider ratio of 1000 to measure the voltage at the pulse capacitor. The stimulation coil is a custom built round coil with a inductance of 16*μ*H.

PLUSPULS is designed to stimulate only if the voltage at the pulse capacitor is in the targeted voltage range. A pulse command will be ignored if this condition is not met. The sequence will be considered to be stable if no pulse commands are ignored during several seconds of stimulation. The missing pulses can be detected with the oscilloscope or by disruption of the normal stimulation sound.

### 7.1 rTMS

**Fig. 9:**
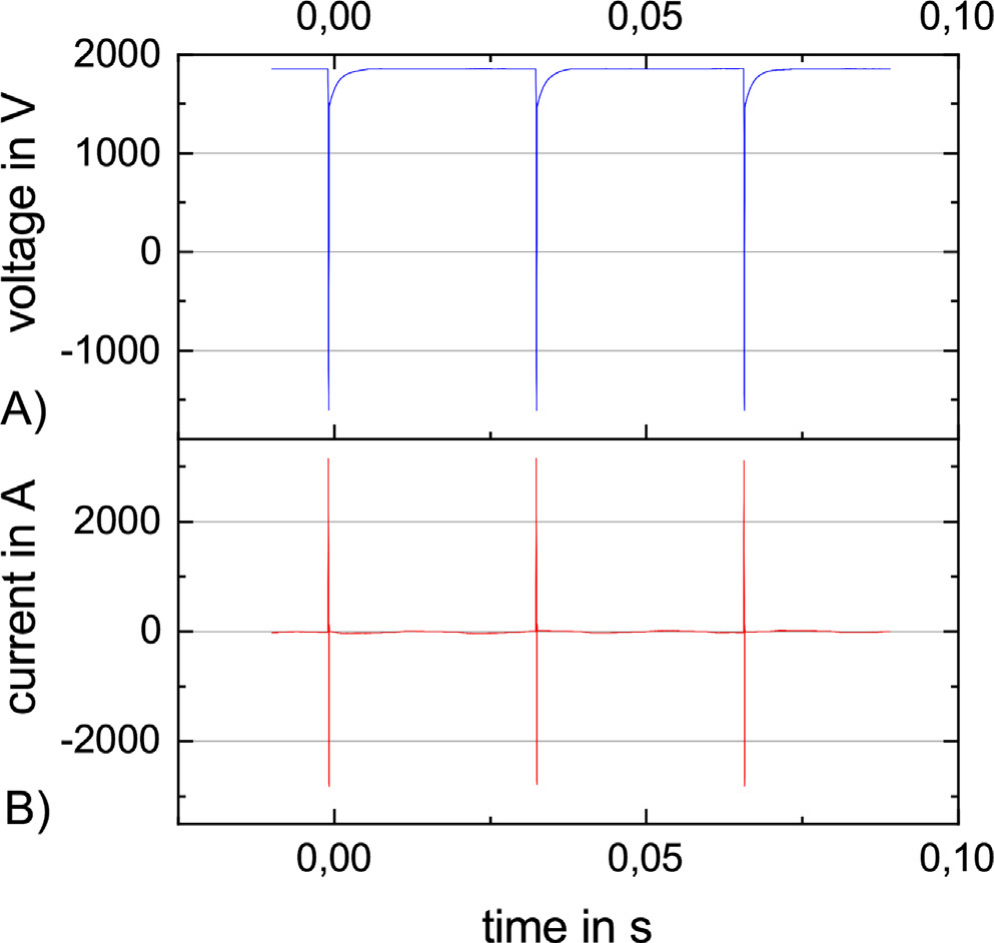
A rTMS sequence at 90% and 30Hz with three stimulation pulses and the following recharge to the target pulse capacitor voltage. (A) The voltage at the pulse capacitor. (B) The current through the stimulation coil.

**Fig. 10:**
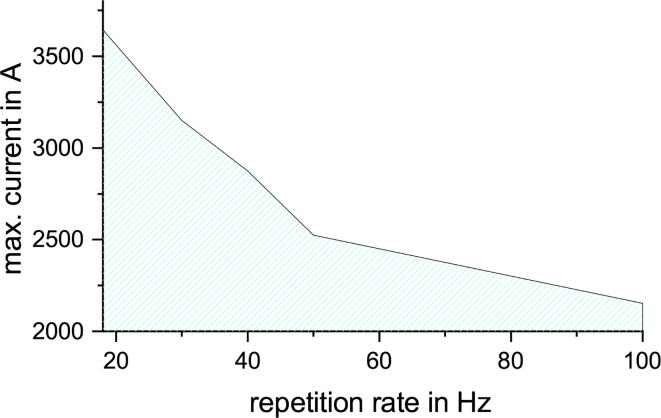
Maximum current amplitude during rTMS stimulation over various repetition rates between 18 - 100Hz.

rTMS is the repetitive stimulation with the same amplitude after the same interval (Fig. 3 A). rTMS can be used for treatment [6] e.g. depression [7]. The characterization with rTMS takes at maximum up to one minute. After one minute, the stimulation coil is likely to heat up to over 40°C, which is the maximum allowed surface temperature because of patient safety. Fig. 9 is with 90% MSO close to the maximum amplitude and with a repetition rate of 30Hz quite fast. The resulting maximum current is slightly bigger than 3000A. Fig. 10 illustrates that with increasing repetition rate the maximum amplitude decreases, starting with 100% MSO at 18Hz and ending with 60% MSO at 100Hz.

### 7.2 ppTMS

The paired pulse (ppTMS) protocol applies two stimulation pulses with a small ISI to one brain region. ppTMS is suitable for treatment and diagnosis. It is the standard to detect ICF or ICI of a neuronal network [4];

It changes for different ISI, with different levels of the first stimuli, the conditioning stimuli [41], and the second stimuli, the test stimuli, between ICI and ICF (Fig. 4).

**Fig. 11:**
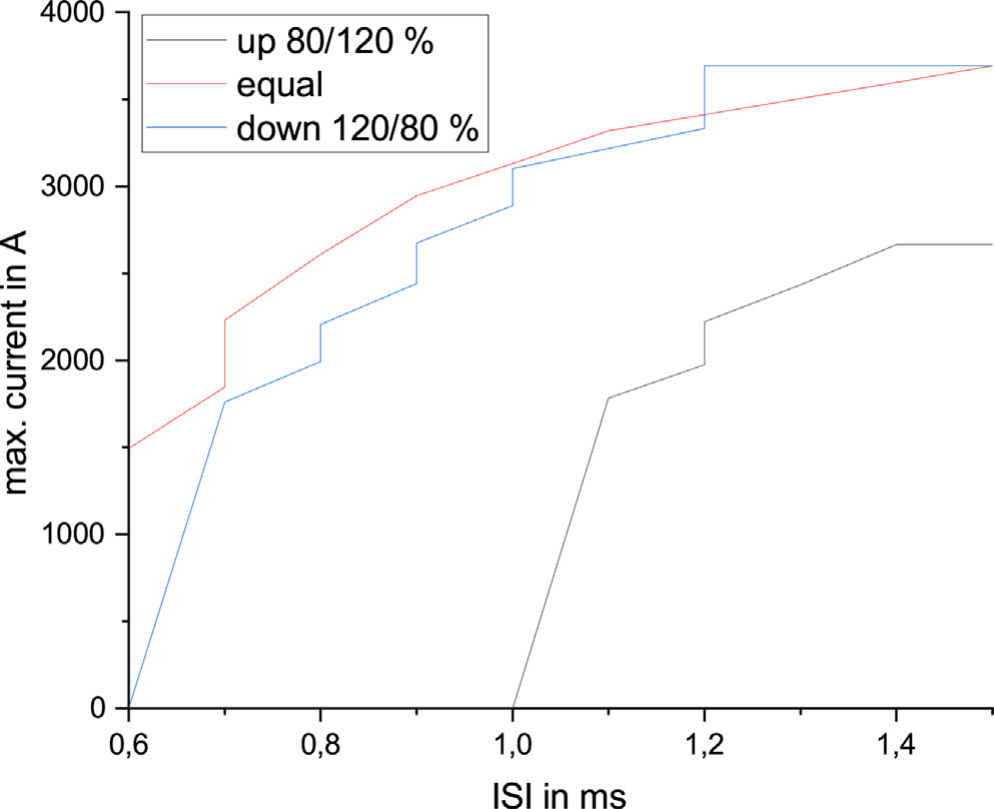
Maximum current of a ppTMS sequence over various ISI and three different ppTMS sequences. The red line represents the limits of the safe operating area (SOA) with a ppTMS sequence with equal amplitudes. The blue line represents the limits of the SOA with a ppTMS sequence with amplitudes around a MT and the first amplitude is at 120% of the MT and the second amplitude at 80%. The difference between blue and black line is the switching of the amplitudes between the first and the second stimulation pulse.

In TMS stimulation amplitudes are in relation to a certain motor threshold. The motor threshold is likely to be in the range of 40 - 60% of the MSO [55], so focus lies on this area. ppTMS is a widely used sequence and is therefore important for the characterization of the design. In all examples the IBI is 1s. The ISI was varied in 0.1ms steps. The ppTMS sequence with unequal stimulation amplitudes are centered around a imaginary motor threshold with a 80% sub- and a 120% supra-threshold pulse. For the equal stimulation the amplitudes is changed in steps of 10% MSO and for the other ppTMS types the motor threshold is adjusted in steps of 5% MSO from 40 - 80% MSO. Fig. 11 illustrates the three different ppTMS sequence types over ISI and the max. possible current of the highest stimulation amplitude.

The ppTMS with equal pulses starts with the lowest ISI for the lowest amplitude. An increase of the ISI results in an increased maximum amplitude. Increasing ISI further results in a diminishing maximum amplitude increase.

The ppTMS with the 80% subthreshold pulse at the beginning (black Fig. 11) needs a higher ISI for the same maximum current compared to the other two sequences.

The ppTMS with the 120% suprathreshold pulse first (blue Fig. 11) has an increased ISI compared to the sequence with equal pulses (red Fig. 11) for low MSO, but decreased ISI for high MSO.

The upper limit of the x-axis of Fig. 11 is 1.5ms. Sequences without a value at the upper limit just copy the highest current value to this ISI. ISIs in steps of 0.1ms without a specific value for this sequence were assigned an interpolated value, if they are in between the measured ISI values.

### 7.3 Quadri Theta Burst Stimulation

**Fig. 12:**
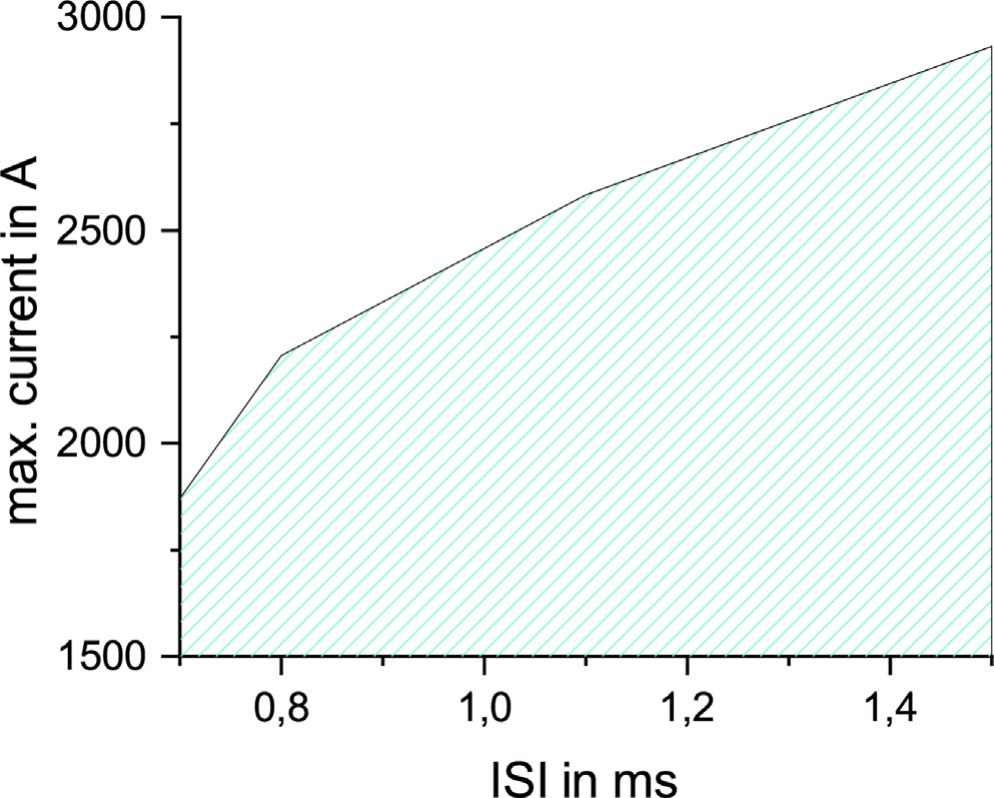
qTBS safe operating area (SOA) for ISI varying from 0.7ms to 1.5ms and amplitudes in a range of 50% MSO to 80% MSO.

PLUSPULS is especially designed for QPS. For this example, we take an IBI of 200ms derived from TBS and combine this to qTBS [20]. A qTBS sequence consists of bursts with four equal stimuli in amplitude and three equal ISI. The ISI is varied in steps of 0.1ms and the amplitude in steps of 10% MSO (Fig. 12).

The maximum current for a specific ISI is lower than for the ppTMS with equal amplitude in Fig. 11. The max. amplitude in Fig. 12 is 80% at 1.5ms. The max. current increases with increased ISI, but with an diminishing effect for higher ISIs (Fig. 12).

#### 7.3.1 Variable ISI

The ISIs have a mean around 1.3ms and the stimulation amplitude is set to 50% MSO. The first one is 1.8ms, the second one is 1.2ms, and the third one is 1.0ms.

**Fig. 13:**
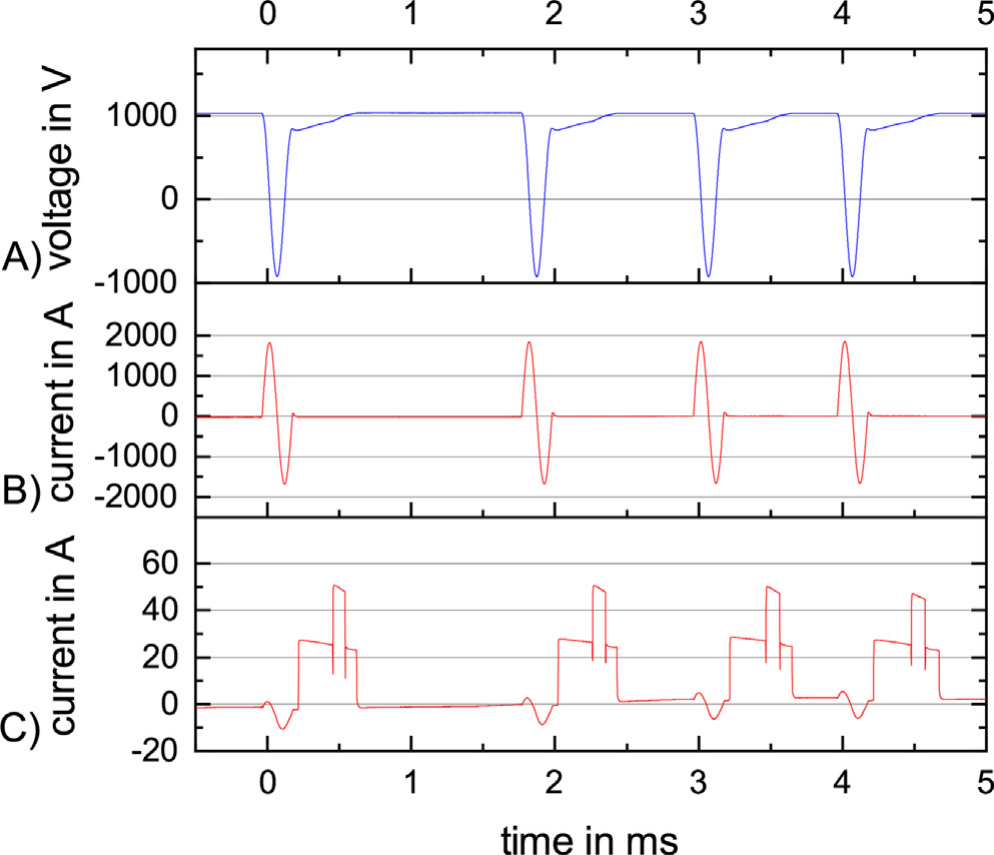
An example of a qTBS sequence with variable ISI and a constant amplitude of 50% MSO, with (A) the voltage at the pulse capacitor, (B) the current through the stimulation coil and (C) the charge current from the high voltage power supply to the pulse capacitor.

The pulse capacitor voltage (Fig. 13 A) increases after the stimulation pulse, the increase is proportional to the charge current (Fig. 13 C). The charge current has several steps in this figure. They are induced by the restriction of the charge current and the two different charge modes, slow and fast. Fast mode is twice as fast as slow mode. Fast mode is used if the pulse capacitor is already charged to 35% of the max. voltage (2720V) of the high voltage power supply to reduce the max. current flow through the charge IGBTs. For low pulse capacitor voltages and for an extended hysteresis around the target voltage, the slow charge is used. This enhances the stability of the pulse capacitor voltage control. In the charge current (Fig. 13 C) artifacts of the stimulation coil current (Fig. 13 B) and a drift can be seen.

#### 7.3.2 Variable ISI and Amplitude

The following example highlights the capabilities of PLUSPULS. The variable ISI selection combined with variable amplitudes of the stimulation pulses in a qTBS sequence is rare and can not be found in a commercial biphasic TMS yet.

The ISIs are taken from Fig. 13 but the amplitudes can vary in a burst. The first amplitude is at 40%, the second at 60%, the third at 70%, and the fourth stimulation pulse at 60% again.

**Fig. 14:**
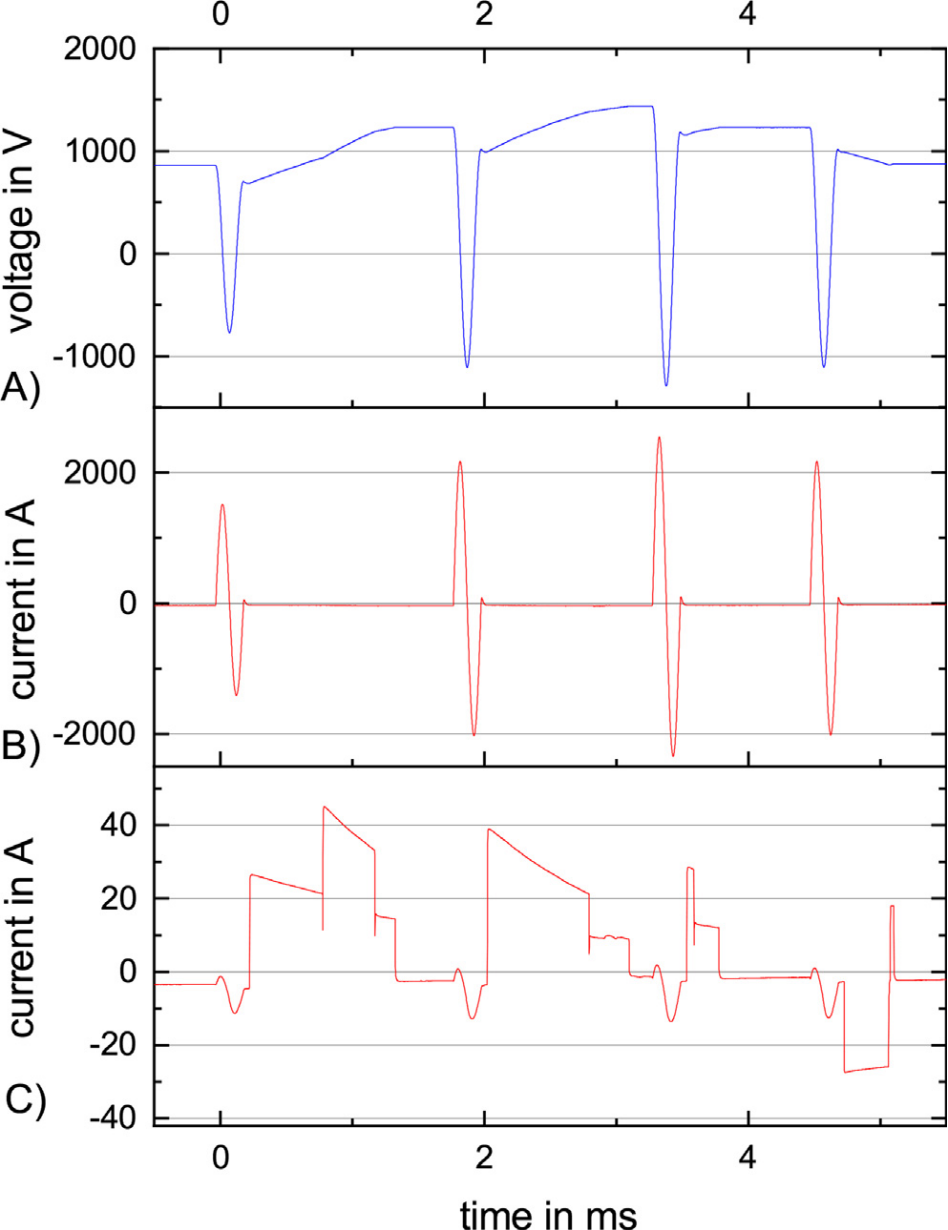
An example of a qTBS sequence with variable ISI and variable stimulation amplitudes of 40%, 60%, 70% and 60% MSO, with (A) the voltage at the pulse capacitor, (B) the current through the stimulation coil and (C) the charge current from the high voltage power supply to the pulse capacitor.

Fig. 14 illustrates the four stimulation pulses with its current (Fig. 14 B) and the corresponding pulse capacitor voltage (Fig. 14 A). The ISI between the second and third pulse is close to the minimum possible ISI which is highlighted by the charge current (Fig. 14 C) becoming zero just before the third pulse. After the fourth pulse, PLUSPULS needs to discharge to the voltage level of the first pulse. As in the previous figures, the charge current has artifacts of the current through the stimulation coil and a drift compared to zero current.

### 7.4 Discussion

The capabilities of PLUSPULS are limited by the maximum supply current from the low voltage system, the charge currents of the pulse capacitors and the quality of the pulse capacitor voltage control.

The high voltage power supply capacitor as an energy storage, with a capacity of 250μF at up to 2720V, provides a small advantage for rTMS and a big advantage for the ppTMS and qTBS burst sequences. rTMS is primarily limited by the power flow. The power flow is limited by the low voltage system, an internal 10A time-lag fuse providing selectivity, which restricts the maximum power in the design, the high voltage power transformer, the high voltage power supply and the charge resistors to the pulse capacitor. During an rTMS sequence the voltage at the pulse capacitor (Fig. 9 A) is charged back to the target voltage long before the next pulse is activated. The maximum voltage at the high voltage power supply is only reached in case of no load. A load reduces the voltage available at the secondary side of the high voltage power transformer. Additionally, the high voltage power supply only recharges if the absolute voltage of the transformer’s secondary side is higher than the voltage at the capacitor of the high voltage power supply, leading to current peaks from the low voltage network around the peaks of the voltage sine wave.

**Fig. 15:**
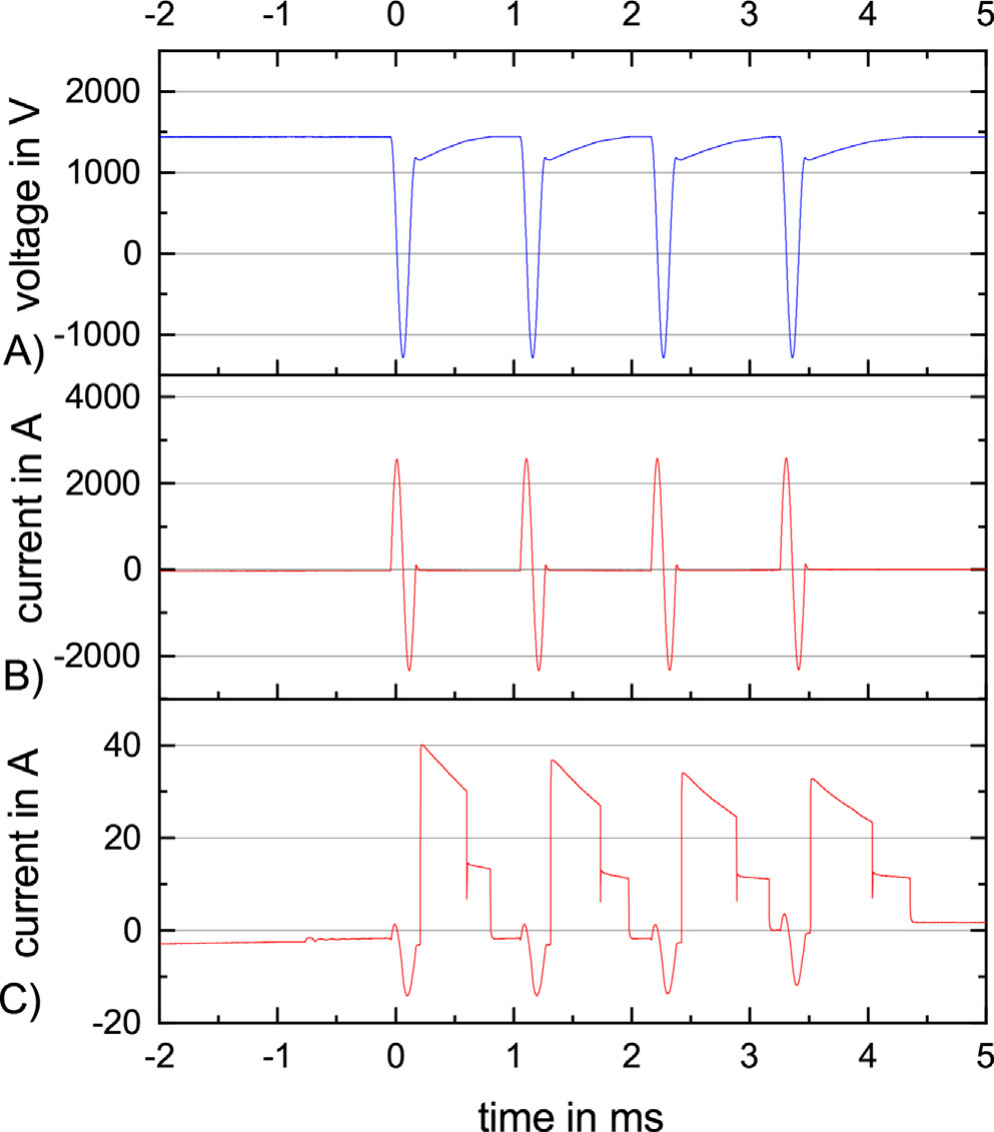
qTBS sequence with constant ISI of 1.1ms and stimulation amplitude of 70% MSO. (A) The voltage at the pulse capacitor just reached the target voltage before the next stimuli. (B) The stimulation pulse current is the same for all four stimuli. (C) The charge current and its maximum decreases and the recharge time increases with the number of stimuli, indicating a decrease of the voltage of the high voltage power supply.

The ppTMS and qTBS sequences are limited by the charge current to the pulse capacitor. The maximum charge current is at the beginning of the recharging after the stimulation pulse, and it decreases with each subsequent stimulation pulse (Fig. 15 C). Assuming that the resistance values of the charge resistors are constant and the losses during the stimulation pulse are the same for all four pulses, only a decrease in the voltage of the high voltage power supply can induce this reduced charge current which also leads to a longer recharge time (Fig. 15 A).

The qTBS sequence in Fig. 15 tests the limits of the charging network. The target voltage for the fourth stimuli is just reached directly before the stimulation and the charge current changes to zero. The charge current has the same drift and stimulation pulse artifacts as in Fig. 13 C and Fig. 14 C.

Another challenge is the stability of the charging and discharging network. The charging has to be fast to enable low ISI for ppTMS and qTBS and leads to a high voltage change rate. The voltage control system has many delays from measurement, digitization, filtering, decision making, and gate driving. This delay adds up to a dead time and can cause instability in the voltage control.

The theoretical maximum voltage change rate is at the maximum voltage of the high voltage power supply, 2720V, and an empty pulse capacitor, with 65μF, charging in fast mode with a 24Ω resistance, which results in a maximum voltage change rate of 1.74V*μ*s^−1^. At the beginning of the PLUSPULS project, the accepted tolerance for the target voltage was  ± 0.5% MSO resulting in a hysteresis window of 22.2V. In this case, the charging system can cross the hysteresis window in around 12.7μs. The delay in the voltage control system has to be lower than the time window needed to cross the target voltage hysteresis window.

This theoretical worst case is prohibited by the software. The fast mode is only possible at high pulse capacitor voltage, and the slow mode has double the resistance values resulting in half the voltage change rate 0.87Vμs^−1^ and double the time needed to pass the hysteresis window (25.4μs).

**Fig. 16:**
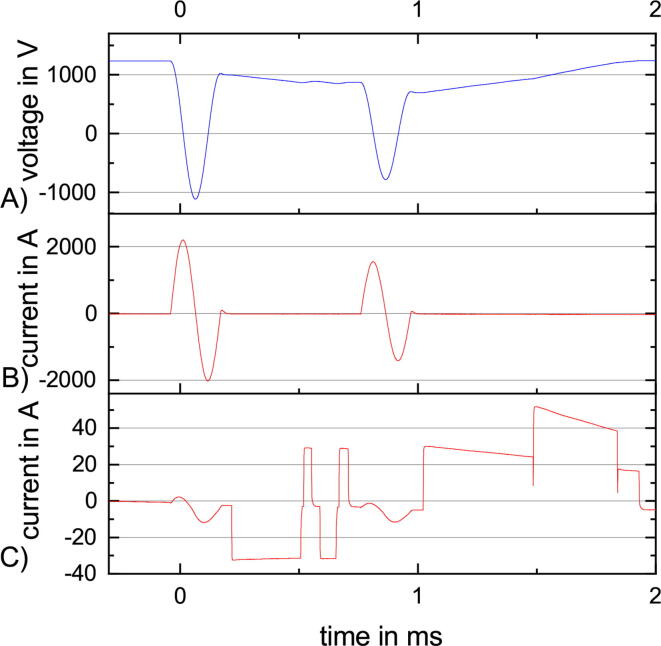
ppTMS sequence with 120% suprathreshold conditioning pulse and 80% test pulse around a 50% MSO motor threshold. (A) The pulse capacitor voltage at 60% and 40% MSO. (B) The stimulation coil current for the two pulse. (C) The charge current is unstable between the first and the second pulse. Artifacts during the pulse, a small offset after the first pulse, and the two different charge modes, fast and slow, after the second pulse can be seen.

**Fig. 33:**
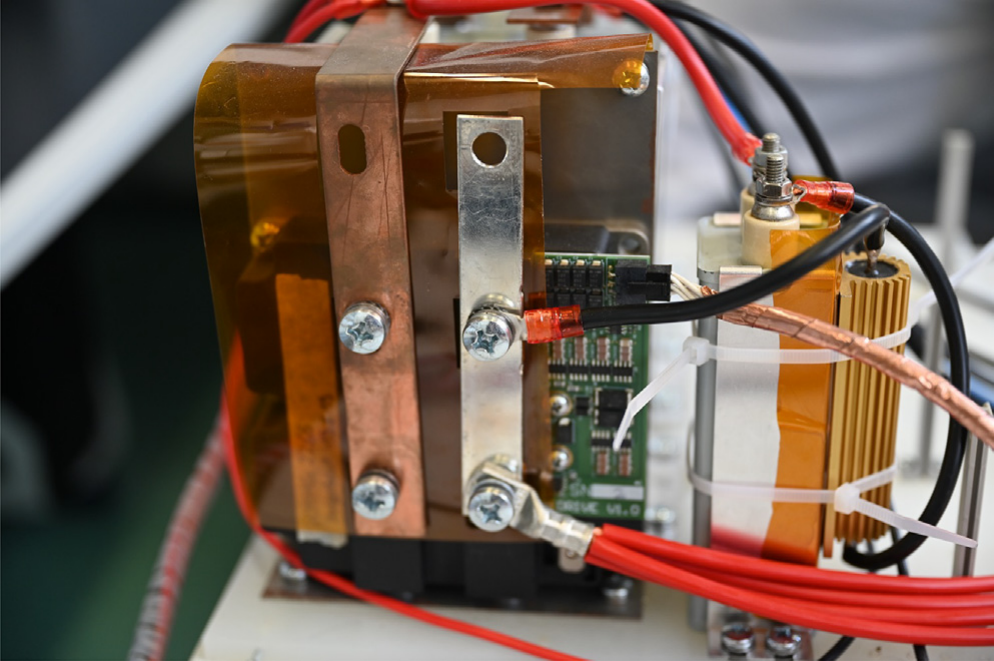
IGBT with IDRIVER screwed to gate and emitter. The ring terminal with the red cables connect the IGBT with the stimulation coil connector. The ring terminal with the black cable connects to the snubber. The copper sheet connects to the positive voltage on the pulse capacitor (6).

The instability of the control system stems from the delay introduced by hardware and software. The internal ADCs of the STM32F415 with a sample rate of 933ksps combined with a sliding mean filter, with width of eight, generate a delay of 8.6*μ*s. The decision to choose either slow or fast charging, idle or discharge is made in the related ADC interrupt. Additional delays are introduced by the driver of the charge IGBT IXEL40N400 and the IGBT itself. The 1090% rise time from discharge to charge or back takes around 12μs (Fig. 16) on top of to the < 2ms delay to switch the gate driver. The stability during discharging is significantly worse than during the charging which results in a high ISI, even for low amplitudes as indicated in Fig. 11. The dead time added to the system by the μC and the control elements is around 22.6ms. The fast mode for charging is disabled close to the target voltage (Fig. 15) and only the slow charge is possible. This results in the dead time 22.6ms being close to the 25.4ms needed to pass the hysteresis window. The measurement circuit also includes a simple RC low pass filter with 1K omega and 18n F, which leads to a tau of 18ms. The RC low pass adds a PT1 element, not a dead time, to the control system.

This means that after overshooting the lower limit and changing to idle state, the voltage is close to the upper limit. Noise and voltage spikes induced by the fast switching of the charge current further decrease the stability of the voltage control.

After the first pulse in Fig. 16, the system needs to discharge the pulse capacitor. After reaching the target voltage, the control circuit decides to switch to charging because of the undershooting of the lower limit. This leads to an alternating charge and discharge decision of the control network which stabilizes after two short charge periods of around 30μs.

To enable a stable voltage control, the hysteresis window was enlarged from  ± 0.5% MSO to -0.5 + 2.5% MSO. This prevents overshooting the target voltage and for the same target voltage, the voltage at the pulse capacitor does not differ as much as this hysteresis window allows (Fig. 15).

### 7.5 Summary of PLUSPULS Capabilities

• rTMS with 90% MSO at 30Hz
• rTMS with 60% MSO at 100Hz
• ppTMS with ISI down to 0.6ms
• ppTMS with variable amplitude
• qTBS with ISI down to 0.7ms
• qTBS with variable ISI and amplitude
• low variation of the voltage around the target voltage
• hysteresis around target voltage of -0.5 - 2.5% MSO
• pulse capacitor voltage up to 2220V at 65m F
• controlled via PC GUI based on Matlab and connected via USB to PLUSPULS
• can add or delete sequence parameters limits in the GUI, like min. ISI for ppTMS depending on stimulus amplitude in % MSO
• connects to 230V low voltage power supply via rubber connector
• internally fused with 10 A time-lag to enable selectivity

*The Deutsche Forschungsgemeinschaft supported the PLUSPULS project with DFG grant proj. No. 398820493. We thank John La Master for proofreading.*

## Declaration of Competing Interest

The authors declare that they have no known competing financial interests or personal relationships that could have appeared to influence the work reported in this paper.

## Figures and Tables

**Fig. 17:**
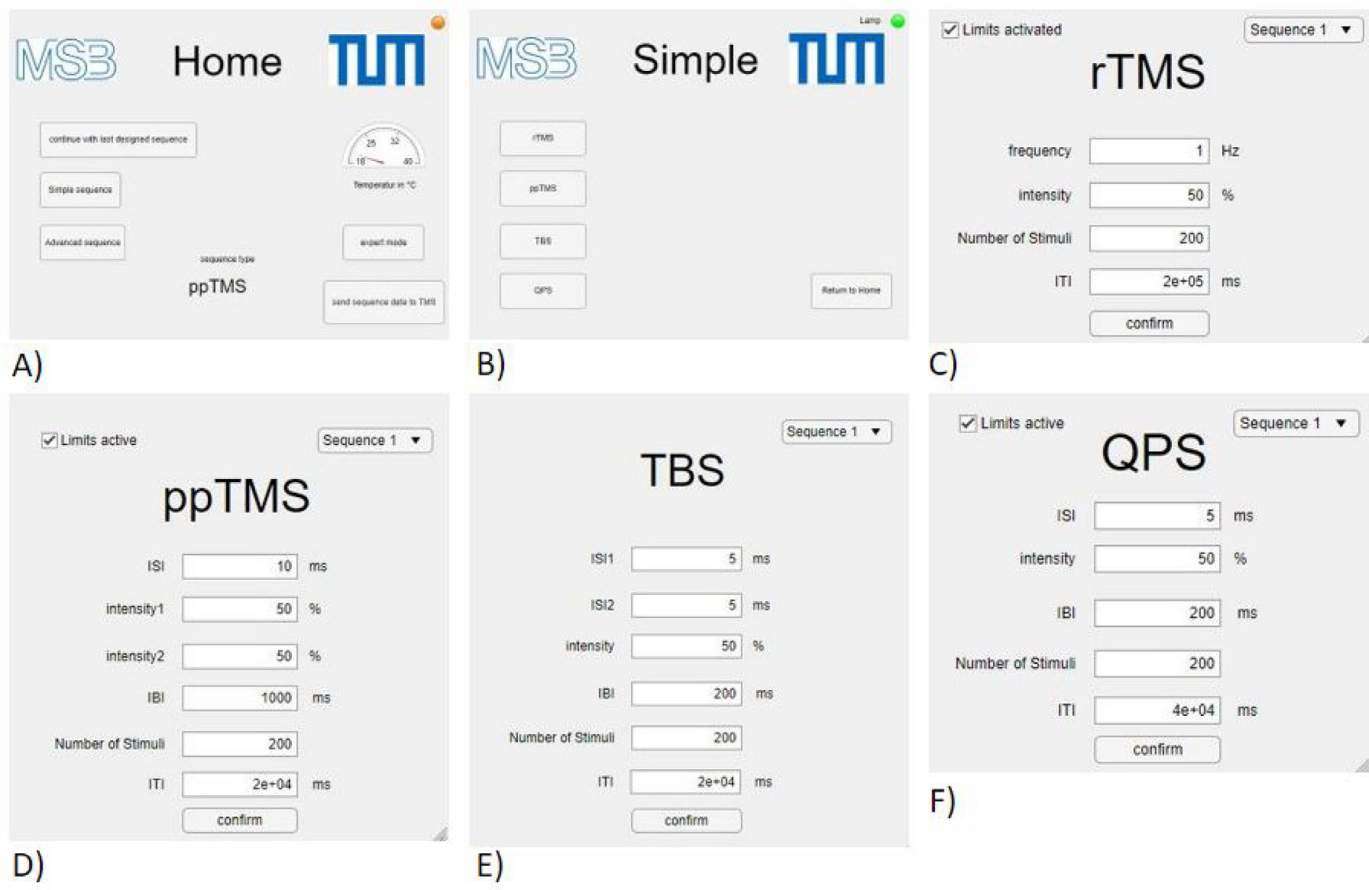
Overview of the Matlab GUI. (A) Home window with buttons for the underlying menus, a lamp at the top right corner for the status of PLUSPULS and the button at the bottom right to send the data to PLUSPULS. (B) Simple menu connects the Home menu to the four different protocol input windows described in (C) to (F). (C) rTMS window to configure the different parameters of the protocol, amplitude/intensity and repetition rate/frequency (D) ppTMS menu to configure the IBI, ISI and the two amplitudes/intensities. (E) TBS menu to configure the amplitude/intensity of all three stimuli. (F) QPS menu to configure the ISI, IBI, and the amplitude/intensity of all four stimuli in one burst.

**Fig. 18:**
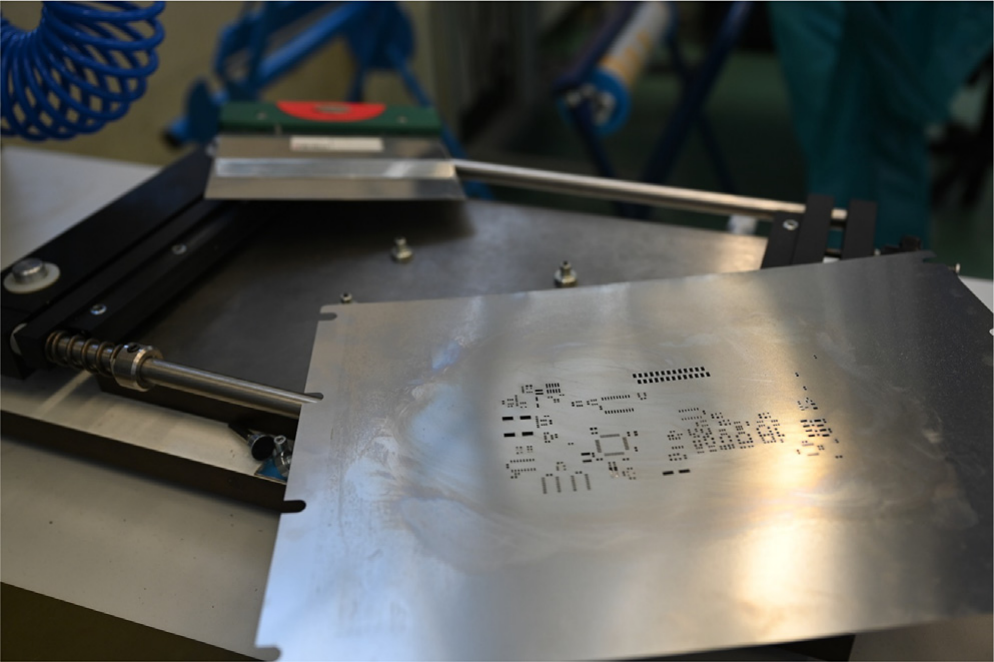
Stencil and spatula located on the stencil holder.

**Fig. 19:**
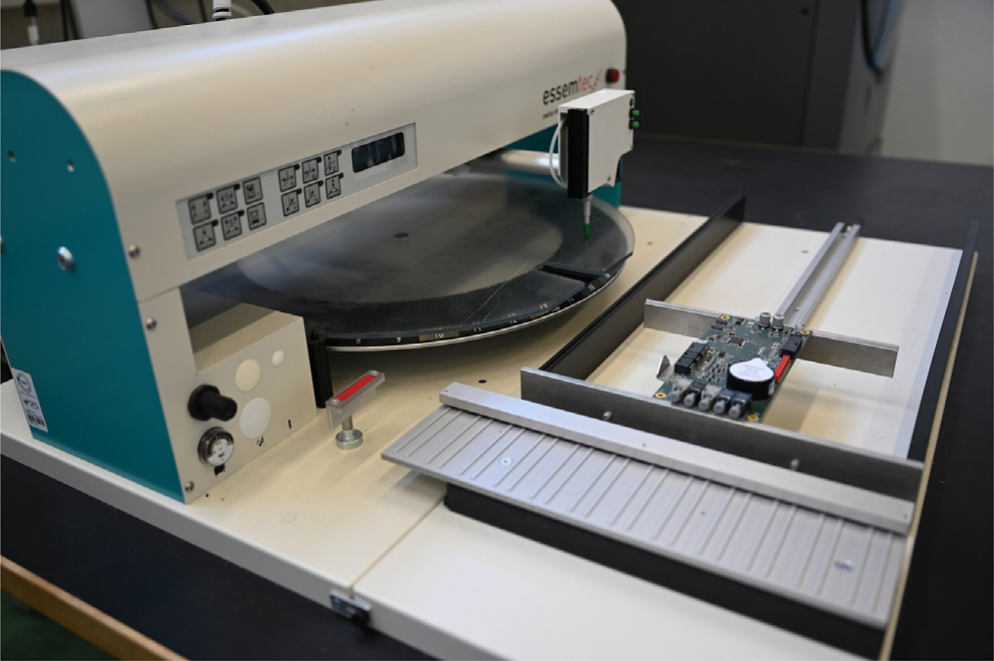
PCB (already finished) placed in front of the semiautomatic tool which reduces hand shaking while placing the SMD components on the PCB.

**Fig. 20:**
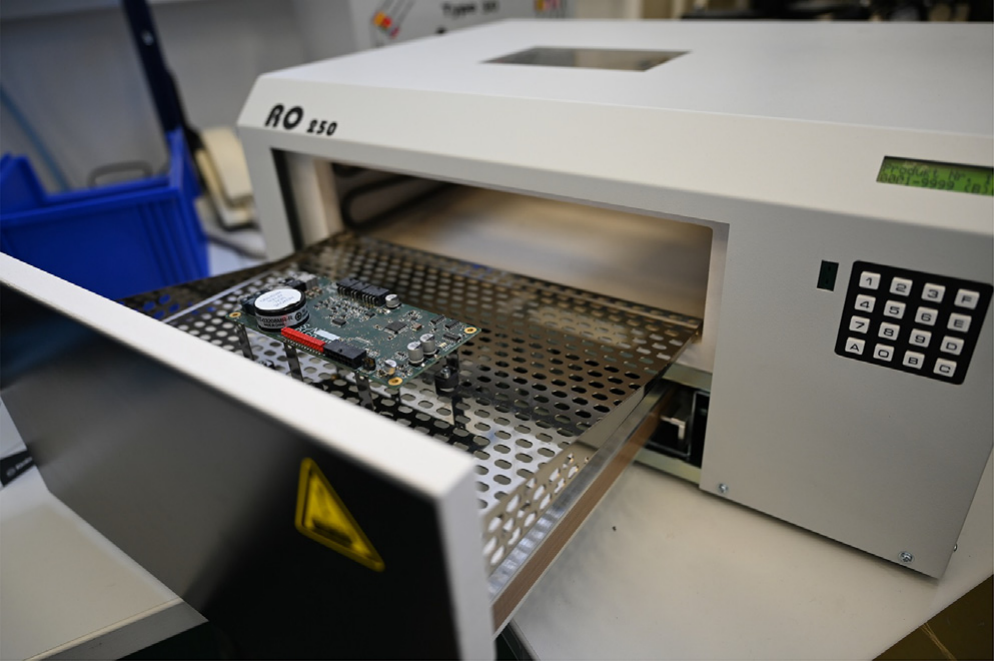
PCB (already finished) placed in the reflow oven ready to start the temperature curve to solder the SMD components on the PCB.

**Fig. 21:**
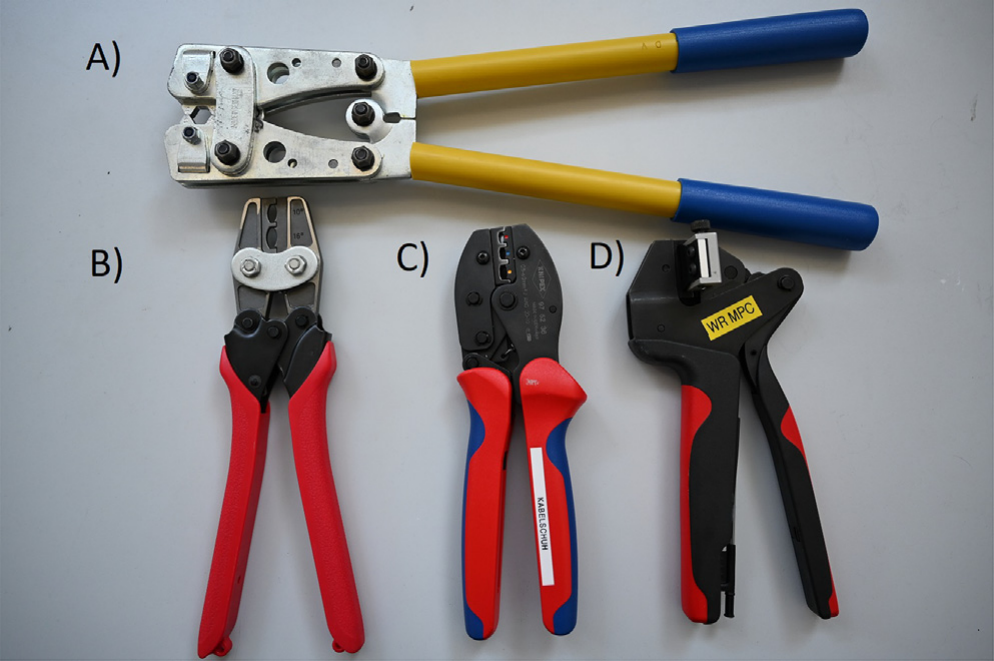
Crimping pliers needed for the plugs. (A) Klauke K05 for crimping the plugs for the Würth RED CUBE connectors and some crimp-on ring terminals for high power connections (B) pliers for 10 - 16mm^2^ crimp-on ring terminals with plastic insulation (C) Knipex 975236 pliers for crimp-on terminals with plastic insulation with cross section between 0.56mm^2^ (D) WE 600 003 pliers with crimping tool 600 662 324 200 (F1 and F2) for pins of the WR MPC3 plug.

**Fig. 22:**
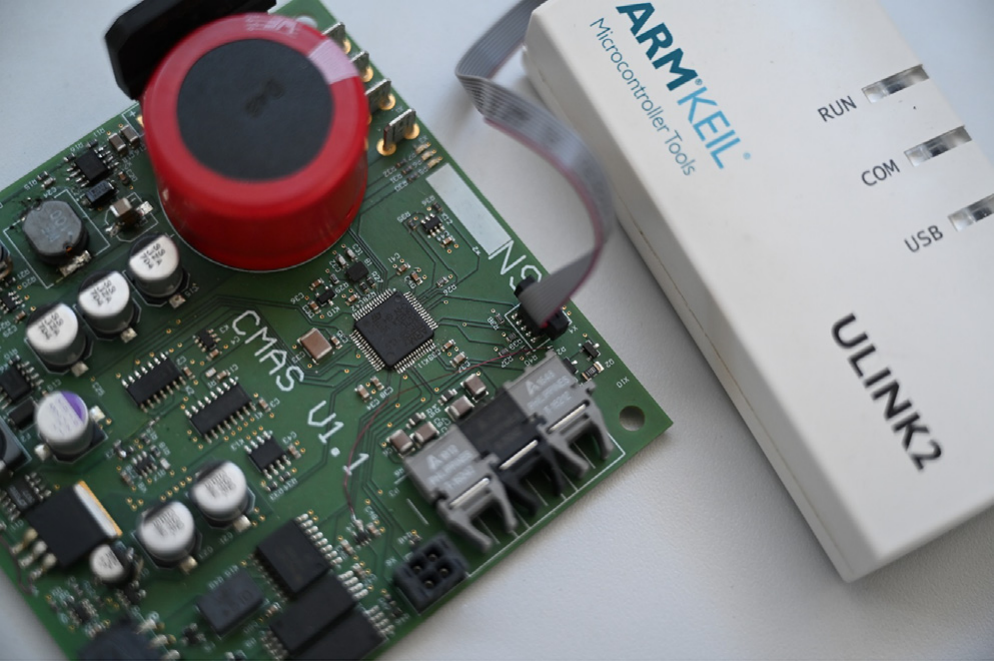
Debugger ULINK Pro connected exemplary to CMAS on the 10 pin JTAG connector.

**Fig. 23:**
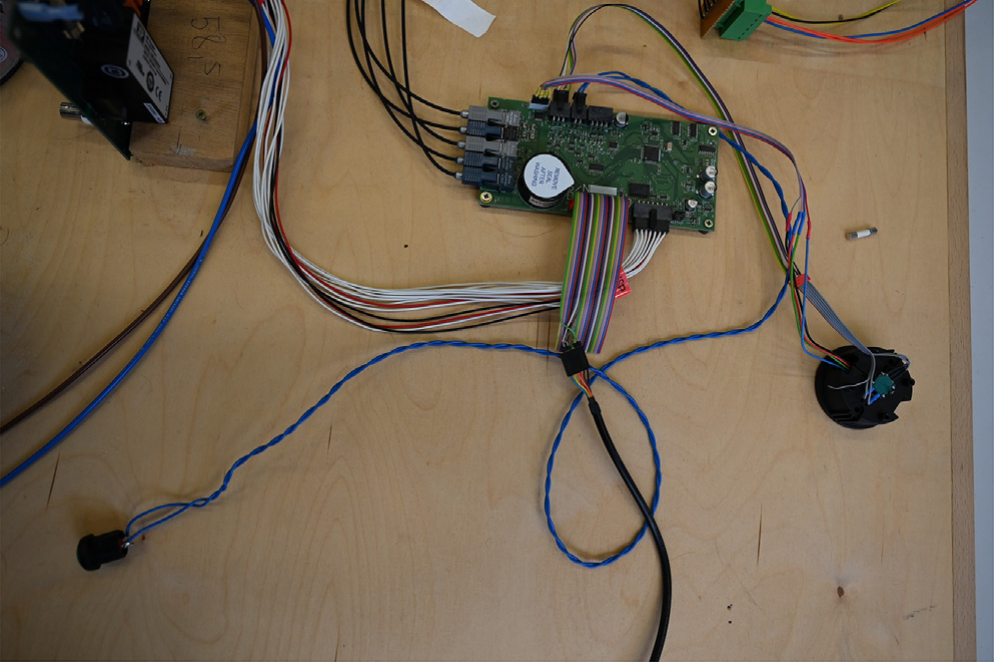
The DISP board with its many connectors. The optical links are used to connect to boards on HV ground potential. The ribbon cable connector was for an optional display and is used for the connection between PLUSPULS and PC. The 14 pin WR MPC3 connector right next to it connects DISP with SISO. The four pin WR MPC3 is used for a push button. The push button switches to ground and needs to be pushed and hold to run a stimulation sequence. On the 6 pin WR MPC3 an optional illuminated push and turn button can be connected to DISP, signalling the state of the PLUSPULS and adding a possibility to adjust values like the stimulation amplitude or ISI.

**Fig. 24:**
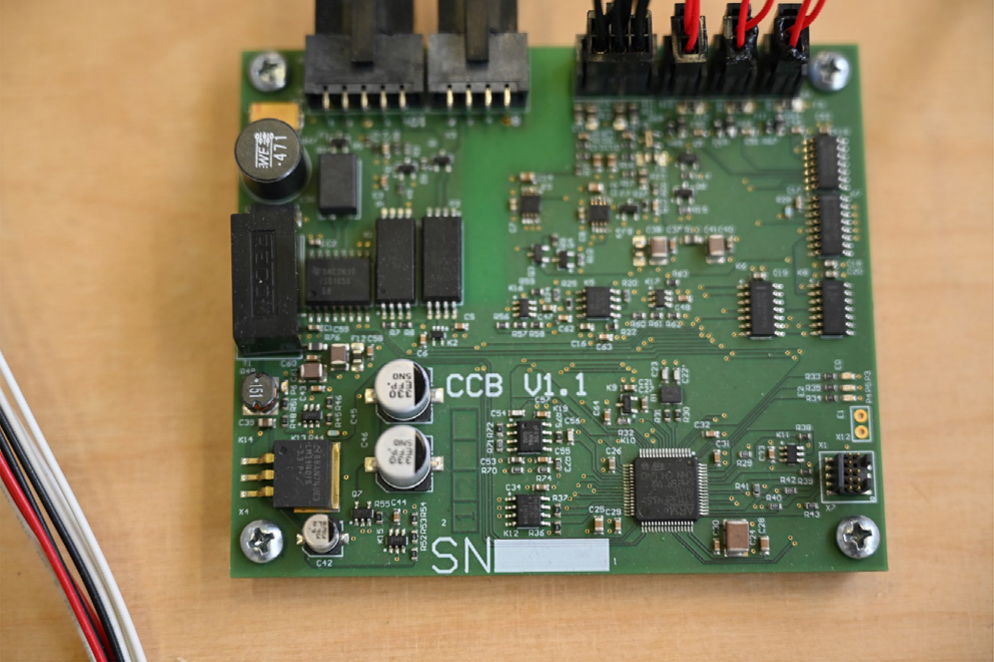
Coil Control Board on HV ground. JTAG connector for flashing and debugging in the bottom right corner, 2 pin WR MPC3 connectors for temperature sensors, 6 pin WR MPC3 connector to connect to the stimulation coil, 10 and 8 pin connectors to connect to the other boards on HV ground via CAN.

**Fig. 25:**
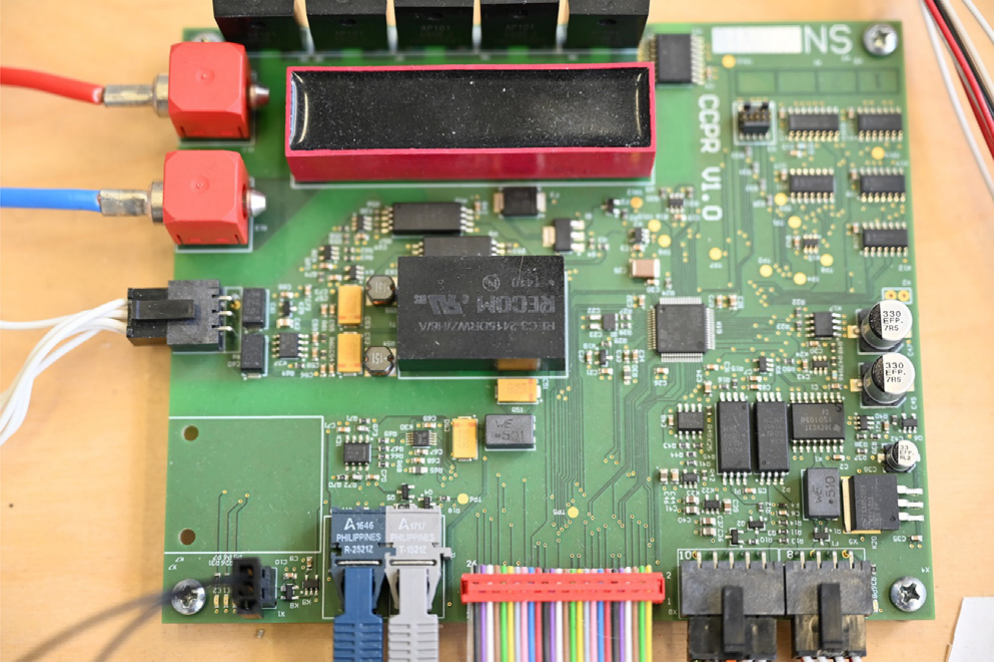
Charge Control Board on HV ground. In the top left corner is the emergency discharge relay with two RED CUBE connectors to the pulse capacitor. At the left side is a 6 pin WR MPC3 to connect CCTRL to IDRIVER. At the bottom side from left to right is a 2 pin WR MPC3 to the foot switch relay on the CPOWER board, two optical links for the trigger in and out signal for the stimulation pulse from and to DISP, the ribbon cable connector to CPOWER, 10 and 8 pin connectors to connect to the other boards on HV ground via CAN. At the top right is the 10 pin JTAG connector for flashing and debugging the μC.

**Fig. 26:**
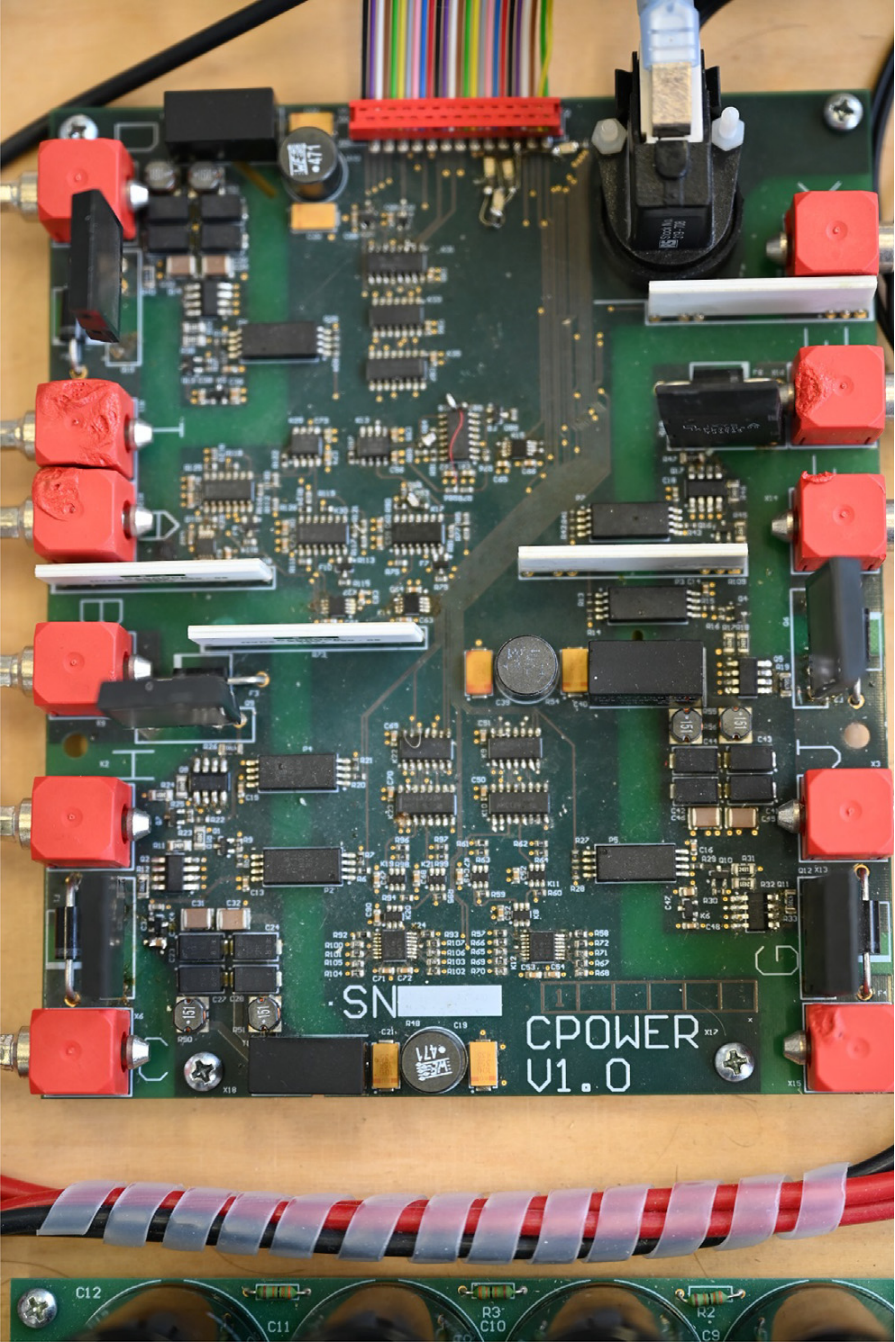
Charge POWER board on HV ground and other potentials. At the top side of the board are the ribbon cable connector to CCTRL and the foot switch relay. On the left and right side are eleven RED cube connectors to connect the charge IGBTs IXEL40N400 to the charge resistors, the high voltage power supply or the pulse capacitor.

**Fig. 27:**
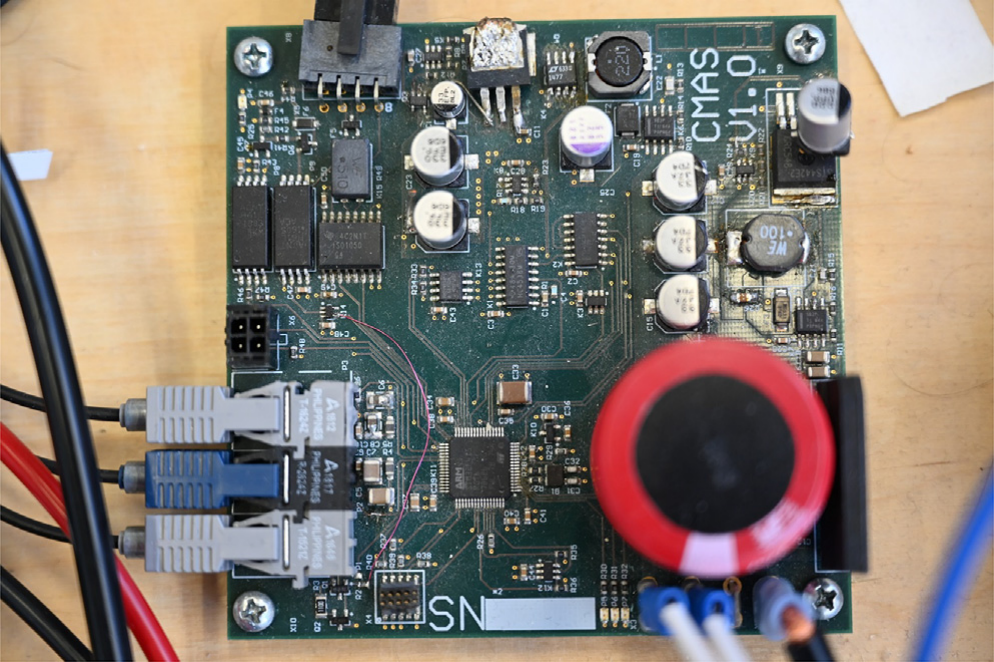
Can MASter board on HV ground. The connector on the top side is the 8 pin WR MPC3 to connect to the other boards on HV ground. The three optical links on the left side of the board are used for the ESTOP signal, the UART send and receive to the DISP board. At the bottom side are the 10 pin JTAG connector for flashing and debugging the μC, two flat plugs to connect the secondary side of the high voltage transformer to CMAS and one flat plug from the HVRECT HV ground potential defining the potential of CMAS and all the other boards connected.

**Fig. 28:**
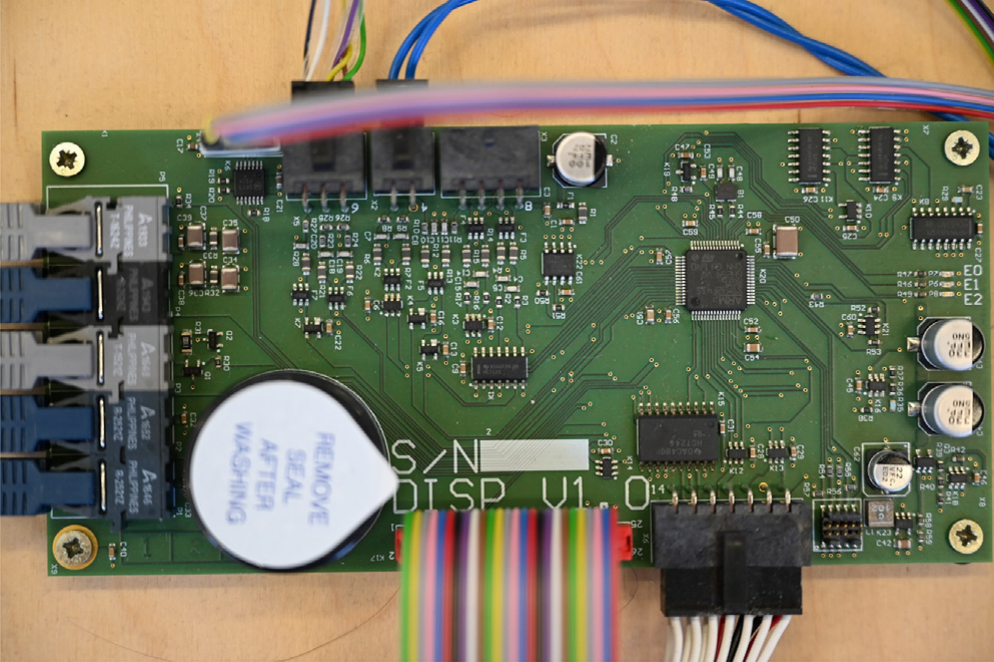
The DISP board with its many connectors. The optical links are used to connect to boards on HV ground potential. The ribbon cable connector was for an optional display and is used for the connection between PLUSPULS and PC.

**Fig. 29:**
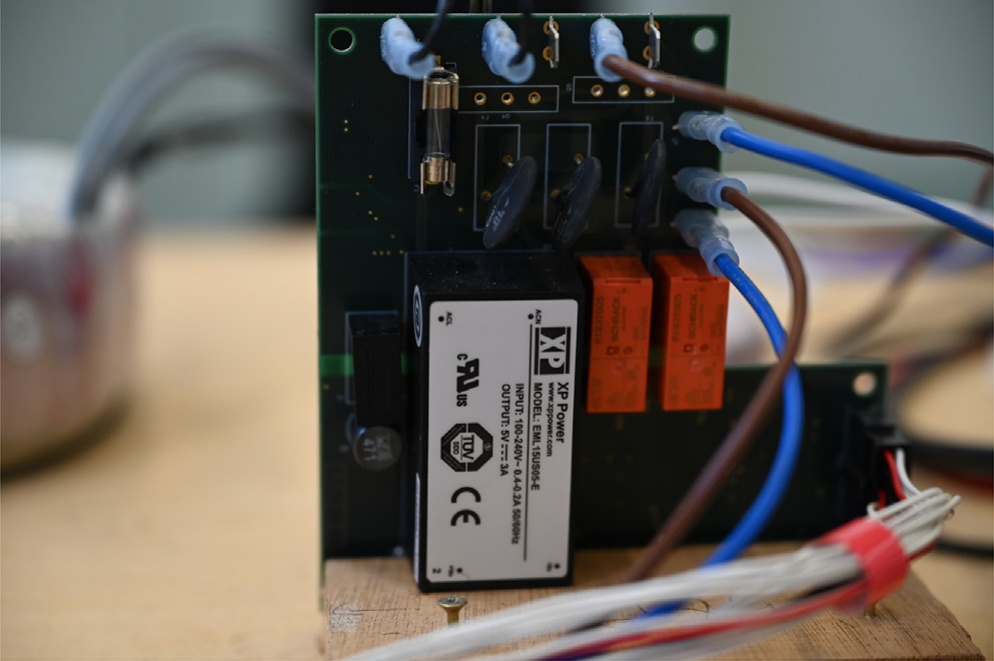
System Input System Output board with the 14 pin WR MPC3 connector to DISP. The two left flat plugs on the top side connect SISO to the primary side of the high voltage transformer and the flat plugs around the top right corner to the primary side of the high voltage power transformer. The other two flat plugs connect the SISO to the 230V inlet of the PLUSPULS.

**Fig. 30:**
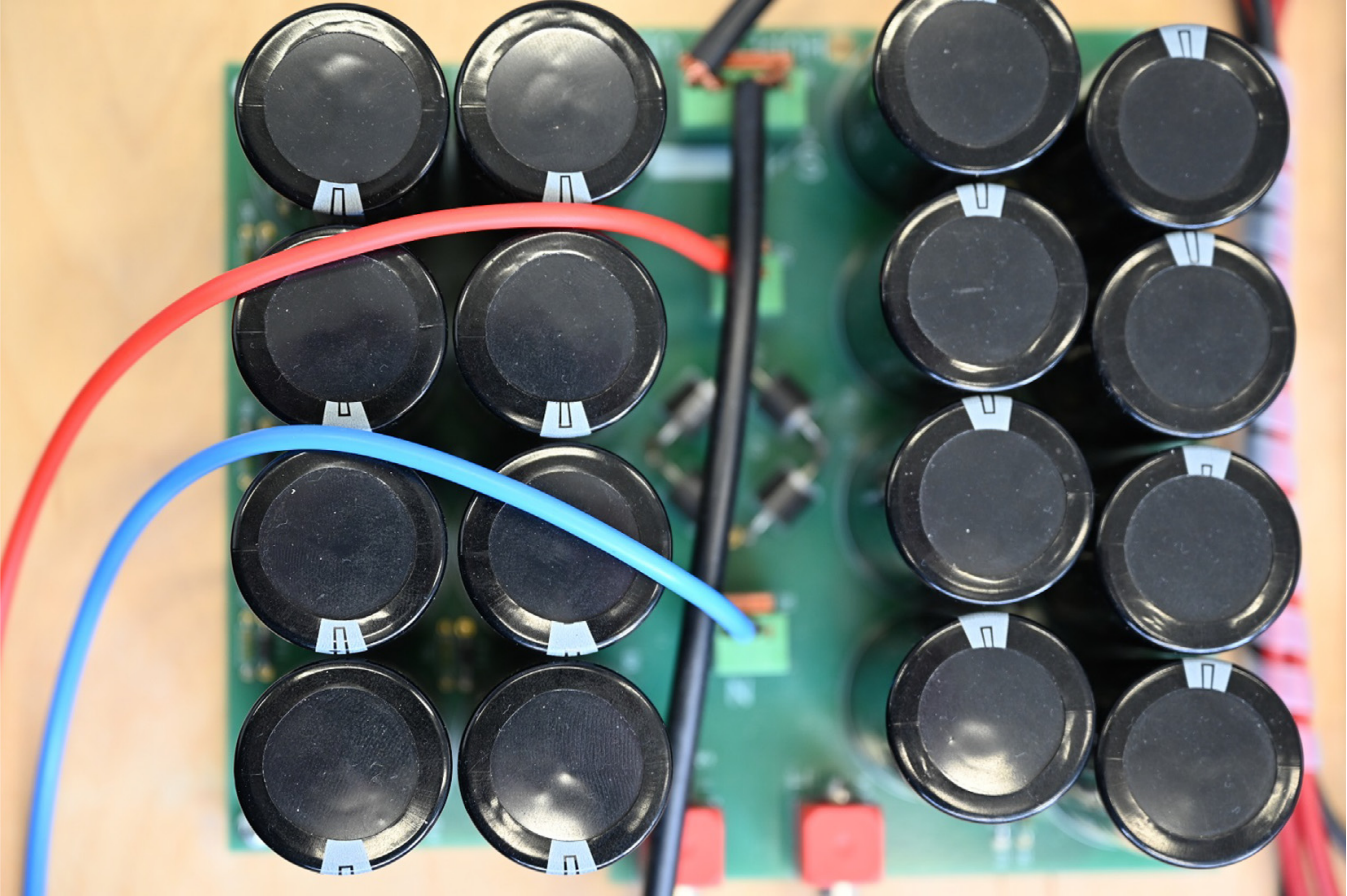
High Voltage RECTifier board with the capacitors for the energy storage and the diodes for the rectification. The HVRECT is connected to the secondary side of the high voltage power transformer with the red a blue cables. The black cables are connected to the CMAS board and to the two symmetry capacitors (C8, C9 Fig. 6) and and provide HV ground potential. The two RED Cube connectors on the bottom side are the positive and negative voltage of the high voltage power supply.

**Fig. 31:**
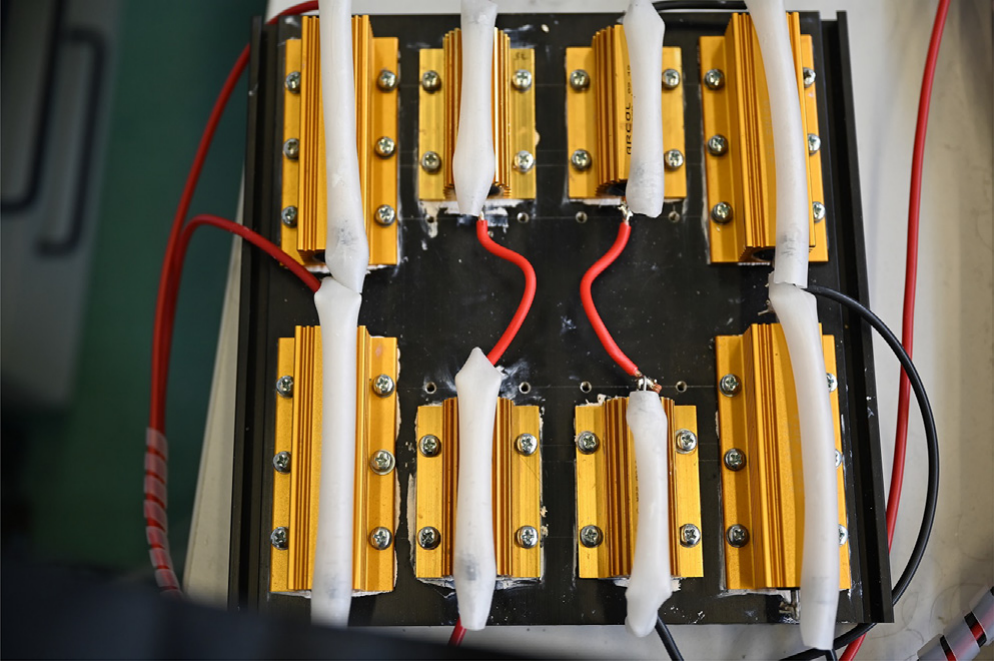
Charging network mounted on a heat sink. The resistors are screwed in customized tapped holes with thermal paste in between. The diodes, inside the insulation tube, are connected to the wire wound resistors in parallel to minimize over-voltages during switch off.

**Fig. 32:**
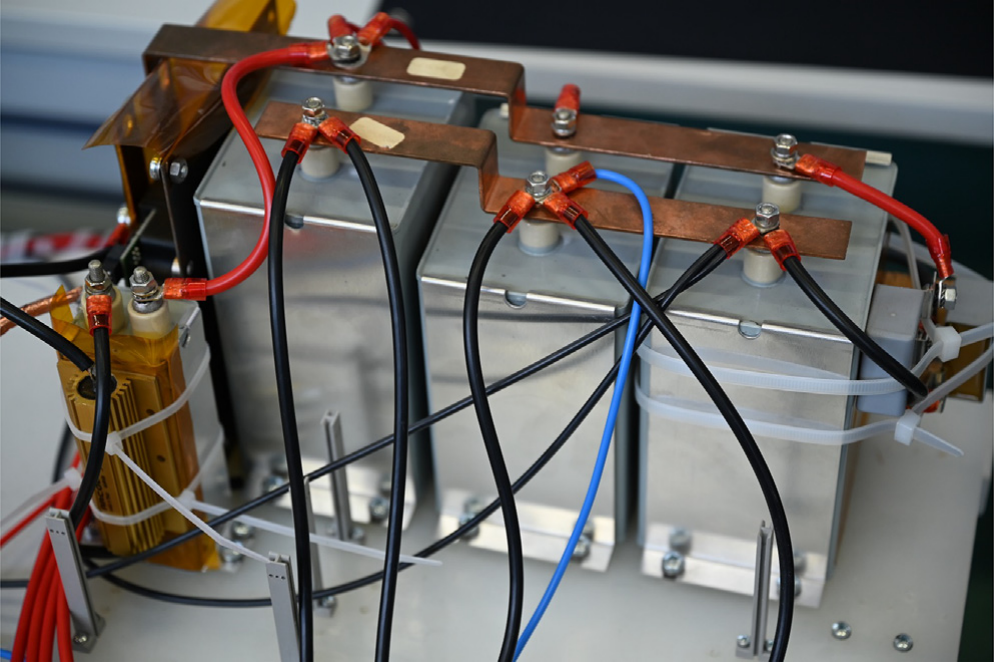
Pulse capacitor made from some three separate capacitors mounted with the IGBT and the corresponding snubber on one board. Additionally two symmetry capacitors (C8, C9 Fig. 6) are mounted on the right side and connected to the positive, negative side of the pulse capacitor voltage and in between to HV ground (Fig. 30).

**Fig. 34:**
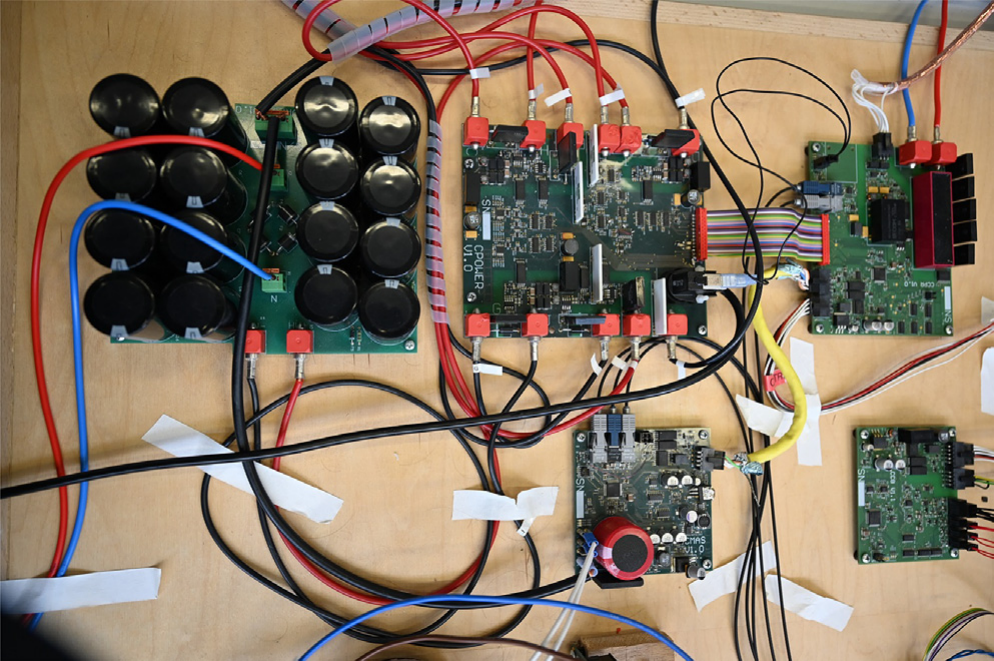
PCBs and interconnections on high voltage ground (HV ground) or connected to it, including the CMAS, CCB, CTRL and CPOWER board.

**Fig. 35:**
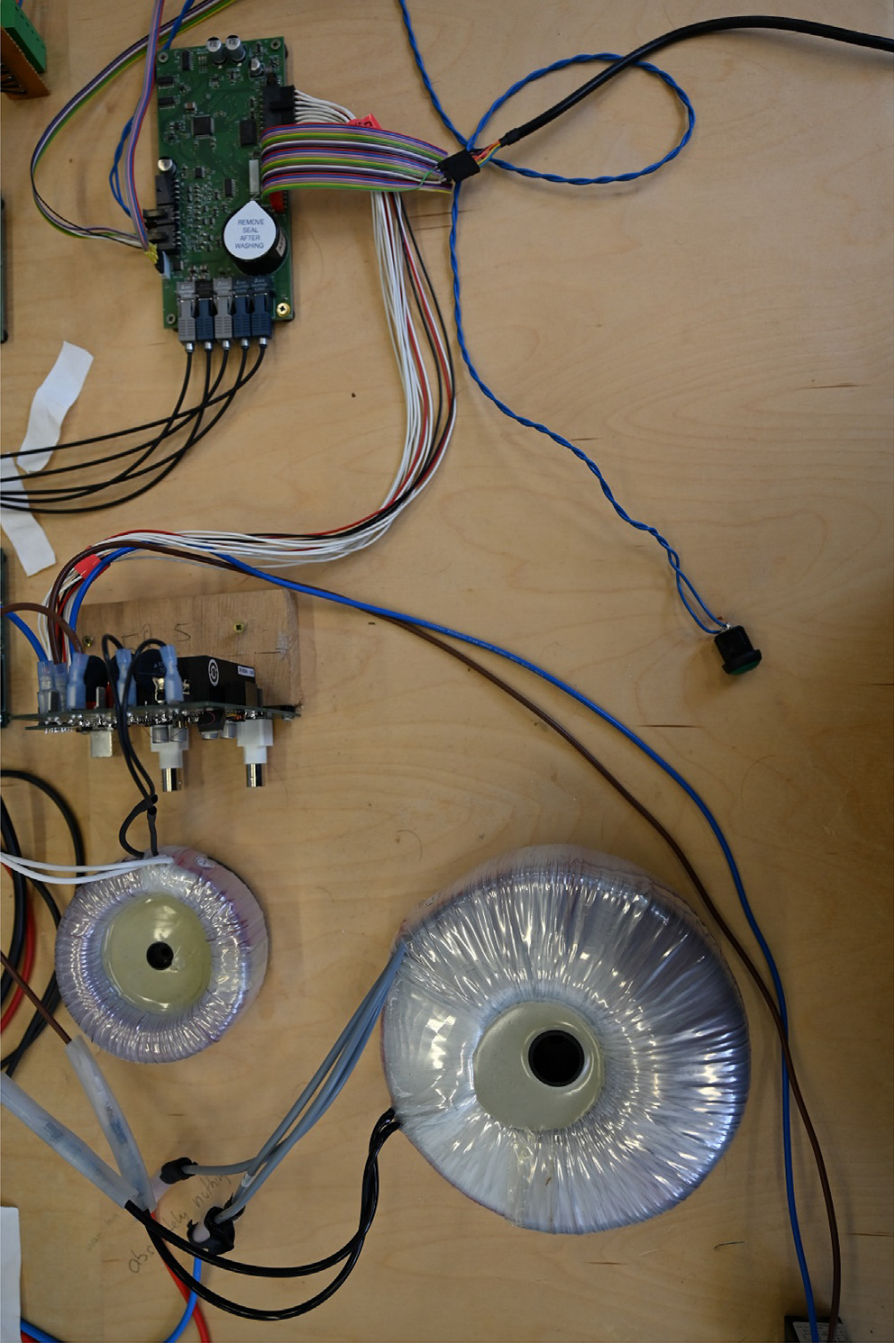
SISO, DISP board and transformers connected to the same potential as the prospective housing.

**Fig. 36:**
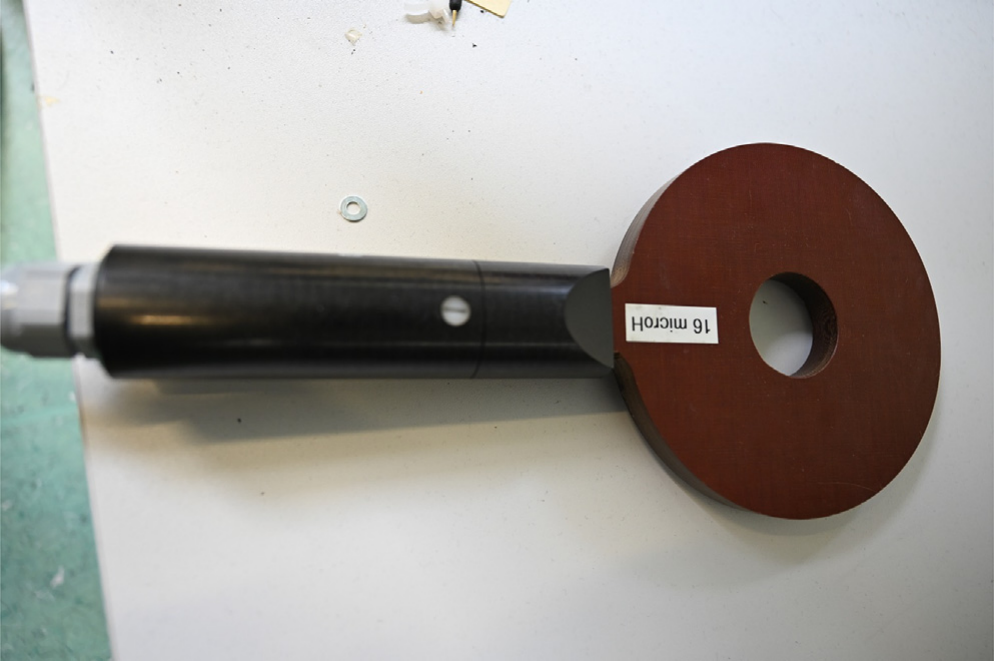
Stimulation coil with inductance of 16*μ*H used for the stimulation.

**Fig. 37:**
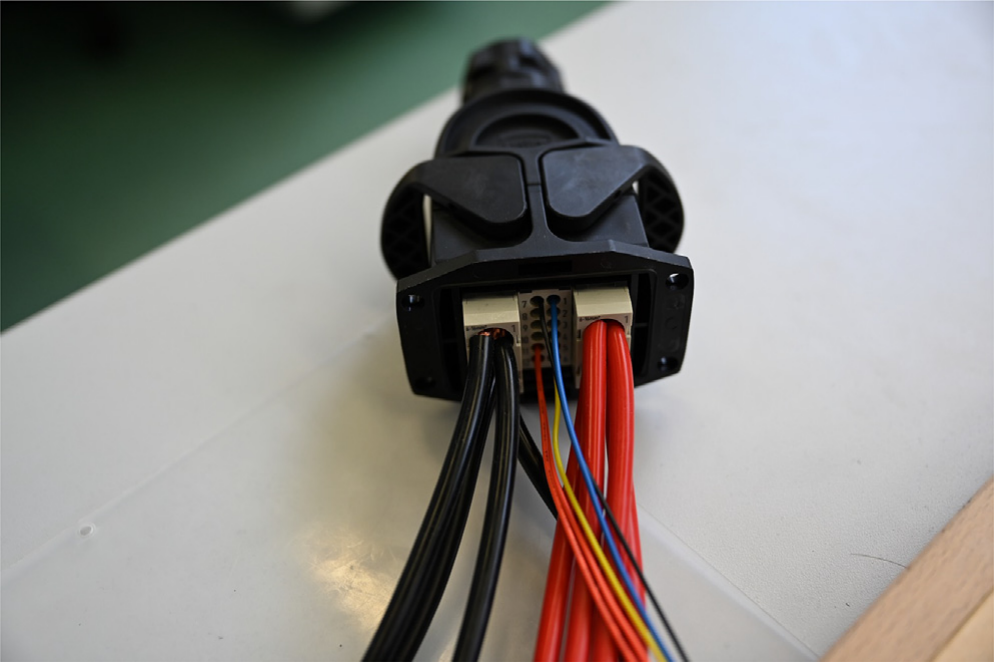
Stimulation coil connector with the two power modules in HAN ECO plug and one signal module in between.

**Fig. 38:**
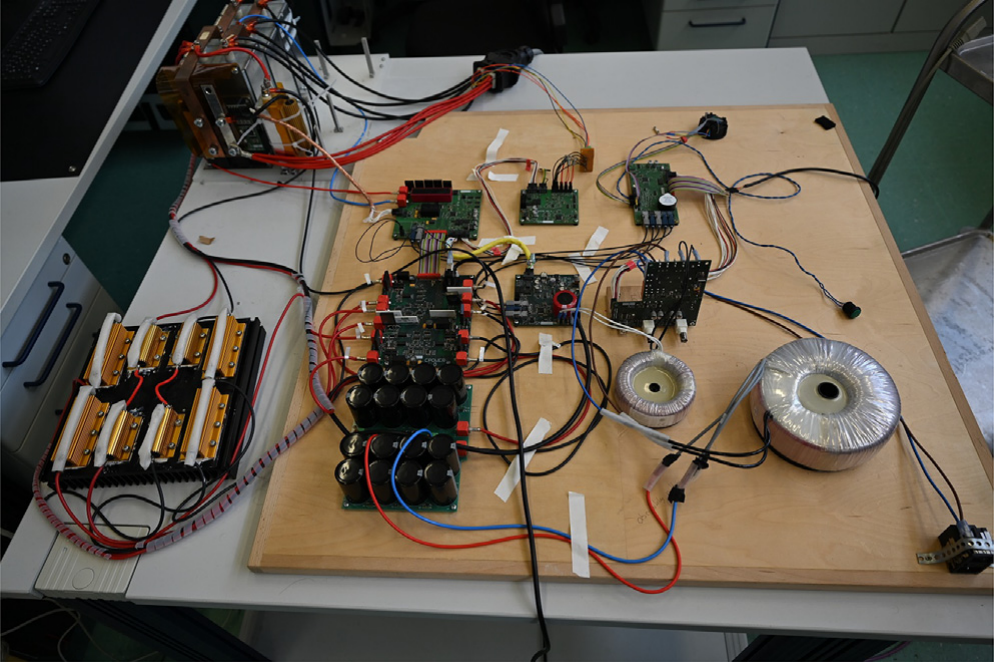
Whole system on a board connected to the charge resistors, the IGBT with the pulse capacitor and coil around the wooden board.

## Appendix A GUI example

.

• Connect the PLUSPULS to the 230 V grid with the help of a rubber connector.
• Connect the stimulation coil to the PLUSPULS and fasten the connector.
• Connect PLUSPULS with Matlab via USB cable to the PC.
• Switch on the PLUSPULS at the main inlet.
• Run the file Home.mlapp in Matlab and wait for the GUI to appear.
• Check the light at the top right corner: Green means the device is ready for programming and in the OP mode. Yellow means the device is not ready. Red means an error occurred.
• Click on the ’Simple sequence’ button, and wait for the new window to open.
• Choose the sequence you want, e.g. pulse TMS (ppTMS).
• Wait for the window to open and rewrite the default and confirm the new values. Confirm all values by leaving the window by clicking ’confirm’. Closing the window should have the same function.
• Close the ’Simple sequence’ window by clicking ’Return to Home’
• The chosen sequence type is visualized in the Home menu.
• If the light is green in the top right corner, press the button ’send sequence data to TMS’.
• Place the stimulation coil at the desired spot.
• Push the pedal button with your foot and press the button at the front plate to start stimulating.
• Closing the Home menu brings PLUSPULS to the PREOP state.

## Appendix B Known Issues

.

• The μC on CCB does sometimes not leave the INIT state. Switch the system OFF and back ON is a known workaround.
• The Matlab GUI should be started after PLUSPULS changes to the PREOP state, otherwise PLUSPULS does not change to the PREOP state after the start.
• Closing the Matlab GUI before shutting down PLUSPULS is advised, otherwise problems can occur when closing the Matlab GUI, which can be resolved by closing Matlab completely.
• The coil temperature measurement is insufficient for coil supervision.

## Appendix C Repository Handling

The repository is on Zenodo and no custom folder structure is possible. The workaround was to zip the files to preserve the folder structure inside.

There are four different types of files. The Matlab files for the GUI, the Altium files for the PCB hardware, the μVision files for the μC software on the PCBs, and the CAD files for the 3D printed parts.

The hardware to construct the PCBs is split in two parts because of upload errors. Both parts Hardware1 and 2 need to be extracted to have access to the complete Altium project group structure. Open the project group in Altium to have access to each PCB project with schematics and layouts. It is also possible to just have a https://www.altium.com/de/altium-designer-viewerviewer. The BOM is also part of each Altium project with the corresponding folder and project structure. It is saved in the folder Project Outputs as a.xls file.

The GUI, based on Matlab Code, needs to be extracted. Start the GUI by double clicking the Home.mlapp file.

The software for all μC is in the.zip file PCB_software. After the extraction, the individual software projects are visible. Open the C code by double clicking the.uvprojx after installing μVision from Keil.

## Appendix D Building instructions

### D.1 Printed Circuit Board Manufaturing

The complete printed circuit board designs as Altium projects are part of the repository. The design rules for example the clearance is part of the projects. A supplier fitting these rules must be found. If the PCBs will be bought including the already placed components the following steps do not apply.

• Buy a corresponding stencil for each PCB fitting your stencil holder. Choose a stencil thickness fitting the smallest pattern in your stencil. 100mm is often a good choice.
• Buy a solder paste, spatula and stencil holder to apply to paste to the PCB.
• Position the PCB under the stencil in the stencil holder.
• Organize your components in a way that allows for a rapid placing of them on the PCB.
• Add the solder paste to the spatula and slide with a angle between 30 and 45 over the stencil with its openings.
• If some pads have no solder paste or are interconnected because of the solder paste, clean the PCB and repeat the last step.
• Open the stencil holder and carry the PCB to your placement area and place the SMD components.
• Open your reflow oven to position your PCB in it. Carry it carefully to the reflow oven. Start a fitting temperature curve for soldering the PCB.
• Check your PCB visually about tombstones etc. Rework errors by hand if needed.
• Solder the THT components to the PCB AFTER the reflow process, otherwise the components will melt during the reflow process.

### D.2 Preparation

Before we start the built some crimping pliers are needed (Fig. 21) for the plugs and pins. (See Fig. 22).

The firmware will be flashed on the μC via the Keil μVision5 IDE and the Ulink Pro or 2 debugger connected to the ten pin JTAG connector. The debugger type should be be found out by the IDE when the debugger connects to the PC.

In order to communicate with PC to the PLUSPULS a TTL-232R-3V3 cable is advised. It has to be connected to the ribbon cable connector X10 (pins 1, 9, 10) on the DISP board (Fig. 28, Fig. 23). The USB connection is converted internally to a UART connection and can be found as a COM port in the device manager. Adjust the COM_port in TMS object in TMS.m as part of the GUI running in Matlab to your COM port shown in the device manager.

### D.3 Images of Boards, Subsystems and the Whole PLUSPULS

In this section the images of PCBs, subsystems and the PLUSPULS can be seen. The CCB (Fig. 24), CCTRL (Fig. 25), CPOWER (Fig. 26), CMAS (Fig. 27), DISP (Fig. 28), SISO (Fig. 29), HVRECT (Fig. 30), DISP (Fig. 28), the charge resistors (Fig. 31), IGBT (Fig. 33), the pulse capacitor (Fig. 32), the PCBs on HV ground (Fig. 34), the PCBs and equipment connected to housing ground (Fig. 35), the stimulation coil (Fig. 35), its connector (Fig. 35) and the whole PLUSPULS (Fig. 35) are listed here. These images used together with the schematics in previous sections should reduce the difficulty to built PLUSPULS.36,37,38.
